# Targeting the muscarinic M1 receptor with a selective, brain-penetrant antagonist to promote remyelination in multiple sclerosis

**DOI:** 10.1073/pnas.2407974121

**Published:** 2024-07-31

**Authors:** Michael M. Poon, Kym I. Lorrain, Karin J. Stebbins, Geraldine C. Edu, Alexander R. Broadhead, Ariana J. Lorenzana, Jeffrey R. Roppe, Jill M. Baccei, Christopher S. Baccei, Austin C. Chen, Ari J. Green, Daniel S. Lorrain, Jonah R. Chan

**Affiliations:** ^a^Contineum Therapeutics, San Diego, CA 92121; ^b^Department of Neurology, Weill Institute for Neurosciences, University of California, San Francisco, CA 94158

**Keywords:** oligodendrocyte, myelination, muscarinic receptors, multiple sclerosis

## Abstract

Multiple sclerosis (MS) is a chronic and debilitating neurological disease that results in inflammatory demyelination. While endogenous remyelination helps to recover function, this restorative process tends to become less efficient over time. Currently, intense efforts aimed at the mechanisms that promote remyelination are being considered promising therapeutic approaches. The M1 muscarinic acetylcholine receptor (M1R) was previously identified as a negative regulator of oligodendrocyte differentiation and myelination. Here, we validate M1R as a target for remyelination by characterizing expression in human and rodent oligodendroglial cells (including those in human MS tissue) using a highly selective M1R probe. As a breakthrough to conventional methodology, we conjugated a fluorophore to a highly M1R selective peptide (MT7) which targets the M1R in the subnanomolar range. This allows for exceptional detection of M1R protein expression in the human CNS. More importantly, we introduce PIPE-307, a brain-penetrant, small-molecule antagonist with favorable drug-like properties that selectively targets M1R. We evaluate PIPE-307 in a series of in vitro and in vivo studies to characterize potency and selectivity for M1R over M2-5R and confirm the sufficiency of blocking this receptor to promote differentiation and remyelination. Further, PIPE-307 displays significant efficacy in the mouse experimental autoimmune encephalomyelitis model of MS as evaluated by quantifying disability, histology, electron microscopy, and visual evoked potentials. Together, these findings support targeting M1R for remyelination and support further development of PIPE-307 for clinical studies.

## Significance

Multiple sclerosis is a chronic and debilitating neurological disease that results in inflammatory demyelination and neuronal loss. Mechanisms that promote remyelination are being considered as promising therapeutic approaches. Previously, the M1 muscarinic acetylcholine receptor (M1R) was identified as a negative regulator of oligodendrocyte differentiation and myelination. In this paper, we describe PIPE-307, an orally bioavailable, brain-penetrant, small-molecule antagonist with drug-like properties that selectively targets M1R. We show that treating cultured oligodendrocyte precursor cells with PIPE-307 leads to their differentiation into myelin-expressing oligodendrocytes. Further, PIPE-307 displays significant efficacy in a mouse model of multiple sclerosis. Together, these findings support targeting M1R for remyelination and encourage further development of PIPE-307 for clinical studies.

Demyelination resulting from diseases such as multiple sclerosis (MS) leads to neuronal dysfunction, namely deficits in signal propagation, subsequent axonal degeneration, and neuronal death. MS is largely driven by myelin destruction (caused by an aberrant inflammatory response) and a failure to efficiently remyelinate. Over time, remyelination becomes less efficient and patients eventually enter a chronic progressive form of the disease due to significant demyelination and concurrent axonal loss (1–5).

Current therapies focus on immunosuppression to limit inflammation and further myelin loss (6). In some cases, demyelinated regions of the brain spontaneously remyelinate, but this process is limited and inefficient, as it typically fails to fully restore lost function. Identifying molecular mechanisms that regulate remyelination is an area of intense interest, and as a result, many promising therapeutic targets have recently emerged (7–10).

Oligodendrocytes are responsible for axonal myelination and are lost during MS. Oligodendrocyte precursor cells (OPCs), while abundant and capable of migrating to lesion sites, fail to differentiate. This may, in part, be due to an inhibitory microenvironment that prevents OPC differentiation into myelinating oligodendrocytes (3, 4, 11–13). Accordingly, increased effort has recently turned toward restoring myelin and subsequent axonal function by triggering innate repair processes, i.e., OPC differentiation and remyelination, for the potential treatment of MS. Using an unbiased screening approach of FDA-approved compounds, two independent groups identified a cluster of antimuscarinic compounds that promoted oligodendrocyte differentiation (7, 8). Benztropine is a well-known antimuscarinic compound used to treat Parkinson’s disease, while clemastine is a first-generation antihistamine with anti-muscarinic activity. While neither compound displays meaningful selectivity against a single muscarinic receptor, using mouse genetic knockouts of each muscarinic receptor, M1 muscarinic acetylcholine receptor (M1R) was implicated as the critical receptor in both clemastine- and benztropine-induced OPC differentiation. Furthermore, targeted genetic deletion of M1R in OPCs was sufficient to accelerate remyelination and reduce the severity of EAE clinical scores (9).

Using a combination of chemical and biological approaches, we further our understanding of M1R antagonism as a promising target for remyelination. First, we identify the protein expression of M1R in OPCs in human and rodent central nervous system (CNS)—including human MS tissue. Importantly, we describe PIPE-307, a potent and highly selective M1R small-molecule antagonist, and highlight its potential for the treatment of MS. PIPE-307 binds with high affinity to M1R and demonstrates exceptional selectivity against a variety of off-targets, including the other muscarinic receptor isoforms. PIPE-307 shows efficacy in several in vitro assays that evaluate OPC differentiation and myelination. Importantly, PIPE-307 was efficacious in the MOG-EAE mouse model of demyelination/remyelination. Our data expand on previous findings that implicate M1R as a target of interest and clearly demonstrate that targeting M1R alone is sufficient to elicit the desired effects in the context of remyelination. Together, these data support a significant role for M1R in remyelination therapies and encourage further development of PIPE-307 for clinical studies.

## Results

### M1R Is Expressed by OPCs.

Despite pharmacological and genetic evidence of M1R on OPCs, unequivocal determination of M1R expression on native OPCs has been elusive. While RNA sequencing databases report M1R RNA expression in OPCs, transcript detection in cells or tissue by RNA in situ hybridization has been challenging due to low transcript abundance. M1R, M3R, and M4R are clearly detectable in OPCs with qPCR and are undetectable in mature oligodendrocytes (9). Moreover, reliable antibodies against M1R or any of the muscarinic receptors have been challenging to optimize or validate for immunostaining (14). As a result, it has been difficult to unambiguously demonstrate that OPCs express M1R.

**Fig. 1.**
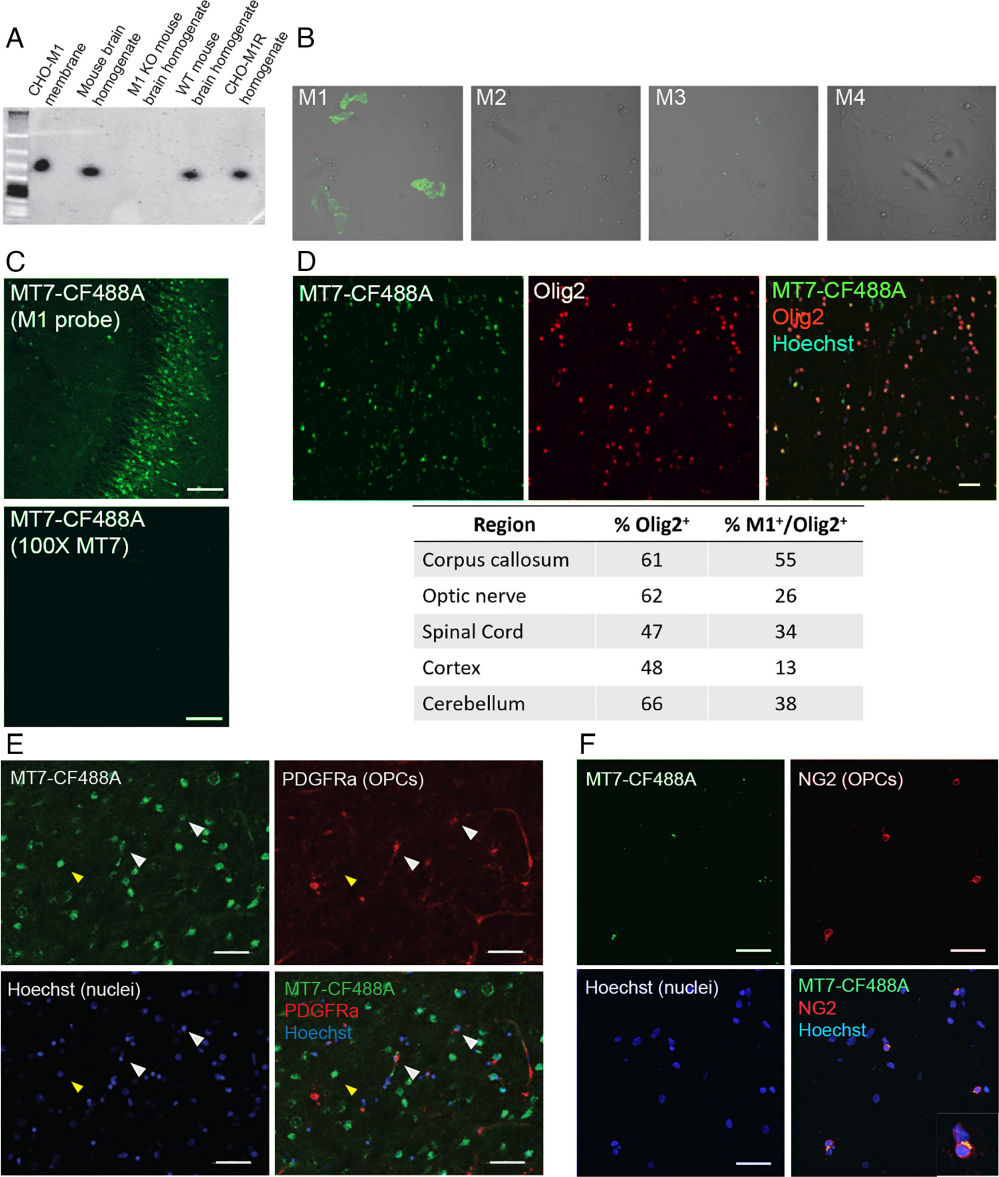
M1R^+^ OPCs are present in rat and human brain tissue sections. (*A*) Lysates from CHO-K1 M1R cells or mouse brain were incubated with MT7-CF488A (M1R probe) and separated by native polyacrylamide gel electrophoresis and fluorescent signal detected. Unbound probe migrates at a low molecular weight, below ladder. Specificity was tested using CHO-K1 hM1R membranes, mouse brain homogenate, M1R knockout (M1R KO) and wild-type (M1R WT) homogenates, and CHO-K1 hM1R cell homogenates. Binding was observed in all samples except the M1R KO homogenates. (*B*) M1R probe (10 nM) binds M1R, but not M2-4R overexpressing CHO-K1 cells. (*C*) M1R probe competition in mouse hippocampal slice, 100× excess unlabeled MT7 blocks probe binding. (Scale bar: 100 µm.) (*D*) M1R^+^/Olig2^+^/Hoechst^+^ cells were quantified in various brain regions using human tissue. Top, representative image from a human corpus callosum section showing M1 probe (green), Olig2 (red), and colocalization with Hoechst counterstain (blue). (Scale bar: 25 µm.) Below, summary table of various human brain regions assessed and percentage of Olig2^+^ and Olig2^+^/M1R^+^ cells. All cells quantified were also Hoechst^+^. (*E*) Fresh frozen rat cortical sections stained for M1R with the M1R probe (green), then immunostained with the OPC marker PDGFRα, and counterstained with Hoechst (blue). M1R^+^ OPC (white arrowheads), M1R^+^ non-OPC (yellow arrowhead). (Scale bar: 50 µm.) (*F*) Human cerebellar brain section stained with M1R probe (green), then immunostained for the OPC marker NG2 (red), and counterstained with Hoechst (blue). The inset is a magnified image of the OPC highlighted with the white arrowhead. (Scale bar: 25 µm.)

Given the limited tools for detecting M1R protein by microscopy, we recognized that a breakthrough to conventional methodology was required. Therefore, we developed fluorescently tagged M1R probes (MT7-CF488A or MT7-CF594) by chemical modification of the M1R selective peptide antagonist, MT7. MT7, a component of green mamba snake venom, displays exquisite selectivity for M1R across species and has shown broad utility as an antagonist of M1R (15–18). Native protein gels were run with membrane preparations from CHO cells overexpressing M1R-5 and brain tissue homogenates. Notably, M1R probe binding was observed in all matrices expressing M1, but not in brain homogenate from the M1R knockout mouse (Fig. 1*A*). Probe binding was observed only in M1R overexpressing CHO cells and could be competed with excess unlabeled MT7 in acute mouse hippocampal slices (Fig. 1 *B* and *C*). The M1R probe was also active and selective in a calcium mobilization assay (*SI Appendix*, Fig. S1).

To gain a better understanding of M1R expression in the brain, we applied the probe in a brain tissue context. We surveyed coexpression of M1R with Olig2 in various brain regions using human brain tissue including white matter tracts such as corpus callosum and optic nerve. M1^+^/Olig2^+^ cells were observed in all regions with the highest percentage seen in the corpus callosum (Fig. 1*D*).

We then combined the M1R probe with fluorescent immunohistochemistry against known OPC markers in mouse (PDGFRα) and human brain tissue (NG2). We observed that the probe showed M1R colocalization with OPCs in both mouse cortical and human cerebellar tissue (Fig. 1 *E* and *F*). Of note, due to a lack of M1R expressing neurons, the cerebellum is a region of low total M1R expression relative to the cortex. Accordingly, the M1R expression we observed in the human cerebellum was primarily in NG2^+^ OPCs and little was seen in NG2^-^ cells (Fig. 1*E*).

**Fig. 2.**
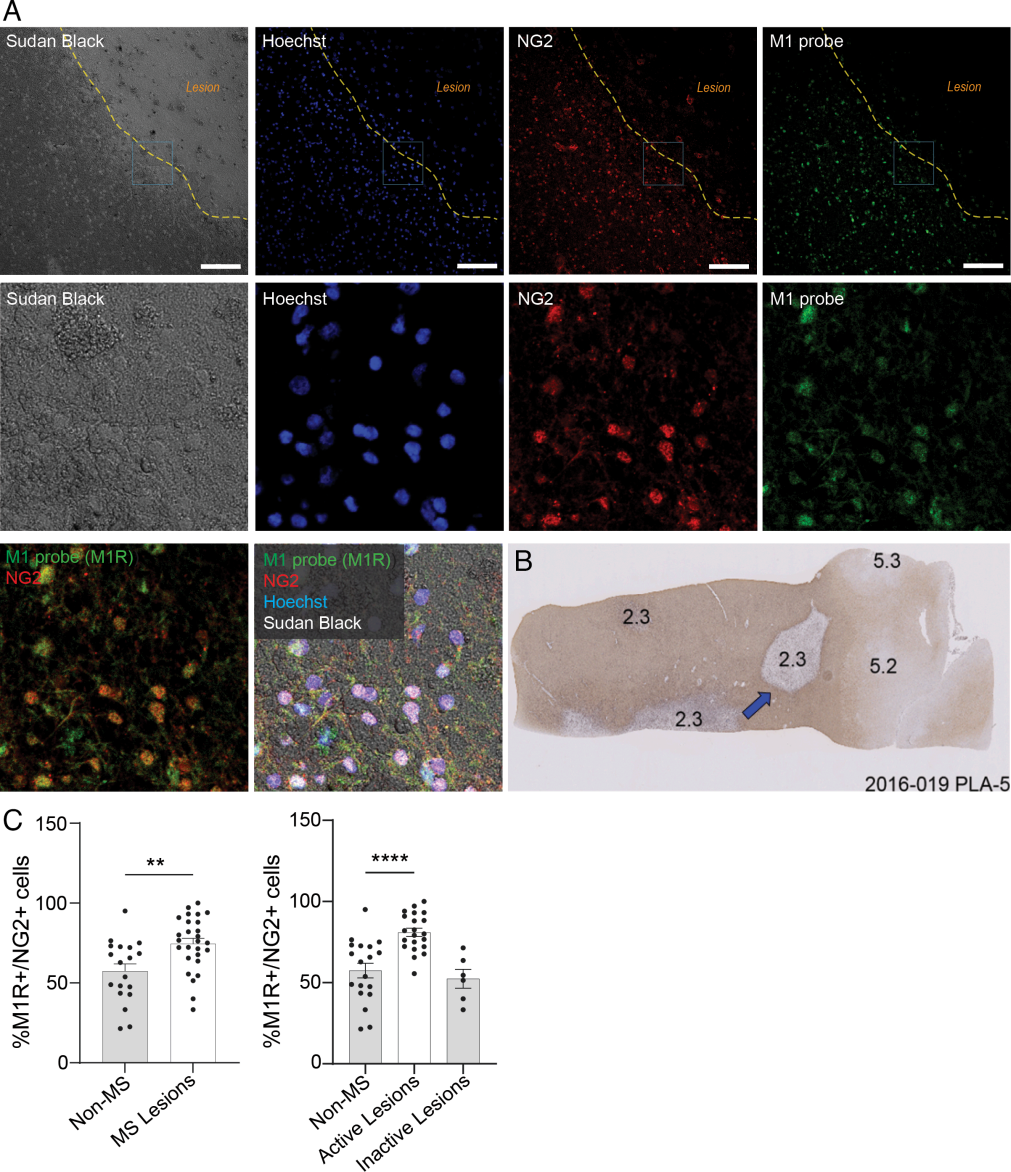
OPCs expressing M1R are found near MS lesions. (*A*) Sections of fresh frozen MS donor tissue from the Netherlands Brain Bank were costained with an antibody against NG2 (OPC marker), the MT7-CF488 (M1R probe), and counterstained with Hoechst. Sections were counterstained with the myelin stain Sudan Black to facilitate lesion identification. The dotted yellow line generally outlines the lesion border. Top row are images at 10× magnification (Scale bar: 100 µm.) The region highlighted in the blue box is magnified in the middle row and bottom rows. (*B*) The blue arrow is approximate region of images highlighted in (*A*). Characterization of the section was provided by the NBB. The lesion was staged as a 2.3 (active demyelinating lesion containing big rounded foamy HLA^+^ microglial cells). (*C*) The percentage of M1R-expressing OPCs (M1R^+^/NG2^+^) in non-MS tissue, in MS lesions, as well as lesions classified as “active“, and in lesions classified as “inactive” were quantified. Lesions were characterized by the Netherlands Brain Bank using previously defined criteria.

To address the critical question of where M1R^+^ OPCs are expressed relative to MS lesions, we obtained fresh frozen human MS donor tissue from the Netherlands Brain Bank (NBB). Lesions were staged by the NBB as defined in the study by Luchetti et al. (19). Here, we characterized a lesion classified as 2.3, an active demyelinating lesion containing activated HLA^+^ microglial cells with foamy morphology throughout the lesion (Fig. 2*B*). Sections were stained using the M1R probe in conjunction with the OPC marker, NG2. Sections were counterstained with the myelin stain Sudan Black, and with Hoechst to facilitate lesion identification. Sudan Black staining revealed a defined lesion border with NG2^+^ OPCs at the lesion border, but few OPCs within the lesion. Importantly, many NG2^+^ OPCs at the lesion border were also positive for M1R (Fig. 2*A*). Next, we quantified and compared the percentage of M1R-expressing OPCs (M1^+^/NG2^+^) in non-MS tissue, in MS lesions classified as “active,” and in lesions classified as “inactive.” The results show that M1R-expressing OPCs are particularly abundant in active MS lesions, implicate M1R as a mechanism for differentiation block, and provide impetus for the development of an M1R antagonist for MS (Fig. 2*C*).

### PIPE-307 Is a Potent and Selective M1R Antagonist.

Previous literature has shown that blocking M1R activity with nonselective muscarinic antagonists or genetically lowering/eliminating M1R expression results in OPC differentiation into functionally competent oligodendrocytes (7–9). Given this, we sought to test whether selective inhibition of M1R with a small-molecule antagonist could recapitulate these data. To this end, we identified and developed PIPE-307, an orally bioavailable, brain-penetrant, M1R selective antagonist.

To demonstrate affinity to the M1 receptor, competitive binding using [^3^H]-N-methylscopolamine as the radioligand was performed with PIPE-307 in membranes overexpressing human M1R, resulting in a K_i_ of 4.6 nM. We also counterscreened PIPE-307 against membranes overexpressing human M2, M3, M4, or M5 receptors and observed >10-fold selectivity against all these receptors (*SI Appendix*, Table S1). We further evaluated functional activity using calcium mobilization in M1R overexpressing CHO-K1 cells. PIPE-307 potently inhibited acetylcholine (ACh) induced calcium mobilization with an IC_50_ of 3.8 nM. A counterscreen using cells overexpressing any one of human M2-5 receptors showed >25-fold functional selectivity against these muscarinic isoforms (*SI Appendix*, Table S1). Together, these data show that PIPE-307 selectively binds and functionally antagonizes the M1 receptor.

**Fig. 3.**
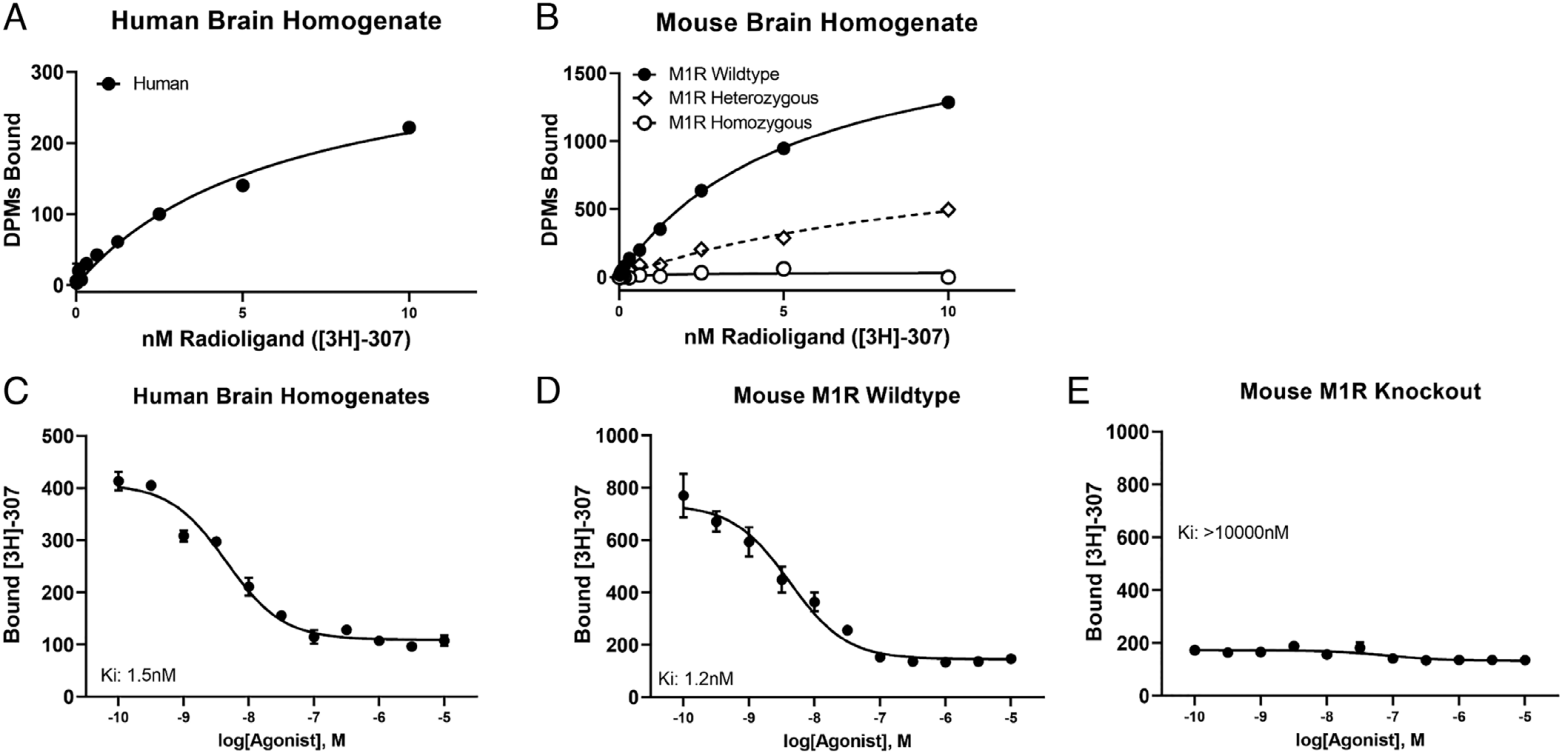
Pharmacological profile of PIPE-307 using [^3^H]-PIPE-307 binding to brain tissue homogenates prepared from adult human cortex and forebrain tissue from adult WT and M1R KO mice. (*A* and *B*) Saturation binding was performed using increasing concentrations of [^3^H]-PIPE-307 in human or mouse brain homogenate. Individual data points represent duplicate samples. (*C*–*E*) Inhibition binding was performed using 3 nM [^3^H]-PIPE-307 with increasing concentrations of cold PIPE-307. Individual data points represent the means ± SEM of n = 4 replicates.

To measure the affinity of PIPE-307 to the M1R, binding experiments were performed in brain tissue homogenates derived from the mouse or human. Using 3 nM radiolabeled PIPE-307 ([^3^H]-PIPE-307), saturation binding in the human brain showed a K_d_ of 6.3 nM and a B_max_ of 348 dpm (Fig. 3*A*). In mouse wild-type tissue, we observed a K_d_ of 5.4 nM and a B_max_ of 1971 dpm, suggesting higher M1R expression in the mouse brain. As expected, the binding of [^3^H]-PIPE-307 was reduced in heterozygous and homozygous knockout mouse brain tissues with a B_max_ of 1025 and 33 dpm, respectively (Fig. 3*B*). It is also worth noting that PIPE-307 had similar potencies in both human and mouse brain tissue. The resulting IC_50_ values were 4.5 and 4.2 nM in the human and mouse, respectively. Adjusting for a radioligand concentration of 3 nM, the K_i_ was calculated to be 1.5 and 1.2 nM for the human and mouse, respectively (Fig. 3 *C* and *D*). Importantly, we observed very little binding in M1R knockout tissue suggesting the total binding is M1R specific (Fig. 3*F* and *SI Appendix*, Table S2).

PIPE-307 was evaluated for potential off-target activities using a panel of 47 targets including G-protein-coupled receptors (GPCRs), nuclear hormone receptors, kinases, ion channels, transporters, and enzymes (Eurofins functional Safety47 panel). Application of 10 µM PIPE-307 did not result in greater than 50% inhibition or activation of any of the nonmuscarinic targets tested (*SI Appendix*, Fig. S2).

### PIPE-307 Promotes OPC Differentiation and Myelination.

An antagonist typically needs the presence of a ligand to show efficacy. Using media conditioned by cultured primary rat OPCs for 3 d, we ascertained the concentration of acetylcholine to be 12 nM (*SI Appendix*, Fig. S3*A*). Since PDGF removal is sufficient to elicit OPC differentiation, we sought to understand the effect of adding exogenous ACh to OPC cultures using this basic differentiation paradigm (20). We observed that adding ACh inhibited OPC differentiation at an IC_50_ of 57.7 nM (*SI Appendix*, Fig. S3*B*). To further understand the dynamics of M1R expression in OPCs, we performed a time course experiment whereby differentiation was initiated by PDGF removal, and the resulting cells fixed at different time points following. Cultures were then stained with antibodies against the following markers: PDGFRα (OPCs), MBP (oligodendrocytes), and the M1R probe (MT7-CF594, *SI Appendix*, Fig. S3*C*). We observed that over the course of differentiation, the population of M1R^+^ /PDGFRα^+^ OPCs decreased, while the number of MBP^+^ oligodendrocytes increased, suggesting differentiation of the M1R^+^ OPC population (*SI Appendix*, Fig. S3*D*).

**Fig. 4.**
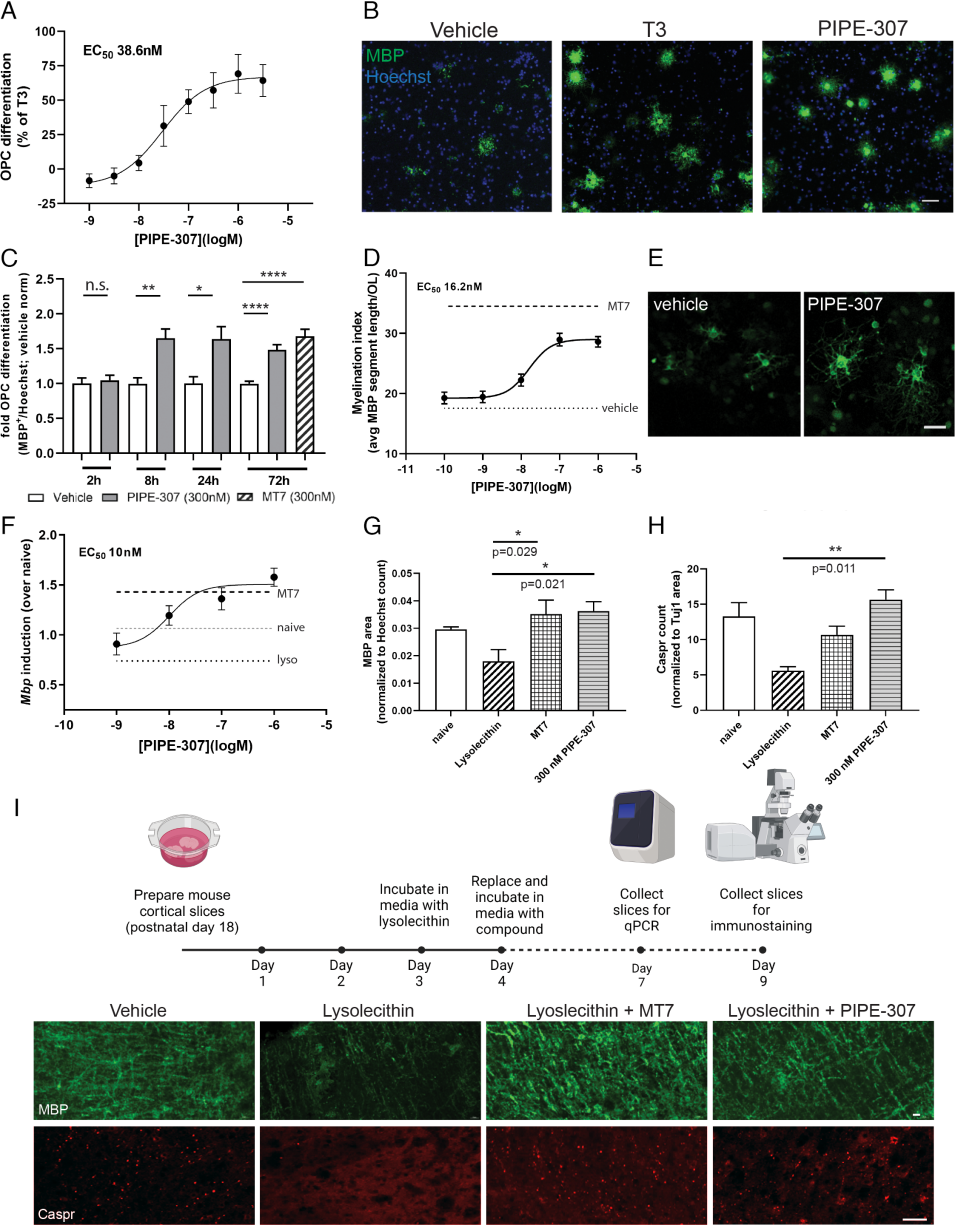
PIPE-307 promotes OPC differentiation and functional remyelination in vitro and in vivo. (*A*) Rat OPCs were cultured and treated with PIPE-307 for 72 h. Results plotted where T3 induced differentiation was set at 100%. PIPE-307 induces differentiation with an EC_50_ of 38.6 nM (means ± SEM, n = 18 wells/data point). (*B*) Images of OPCs treated with 50 ng/mL T3 or 1 µM PIPE-307, MBP (green), Hoechst (blue) (Scale bar: 25 µm.) (*C*) Rat OPCs were treated with 300 nM PIPE-307 for times indicated then OPCs differentiated until 72 h. White bars indicate vehicle, gray bars PIPE-307 treated, and hatched MT7 treated. PIPE-307 values were normalized to vehicle at respective time point. MT7 was included as a positive control (means ± SD, *t* test at each time point, n = 5 per point, 2 h: *P* = 0.65; 8 h: *P* = 0.003, 24 h: *P* = 0.01, 8 h: *P* = 0.0001 for both PIPE-307 and MT7). (*D*) OPCs differentiated with PIPE-307 wrap axons using a cortical myelination assay. Cultures were immunostained with MBP and myelin segments for each oligodendrocyte measured (myelination index) (means ± SEM, n ≥ 38 cells/data point). (*E*) Representative image, MBP (green). (Scale bar: 25 µm.) (*F*) Slice cultures were demyelinated with lysolecithin (Lyso) and then treated with different concentrations of PIPE-307; 300 nM MT7 was used as a positive control. PIPE-307 induces *Mbp* RNA (measured by qPCR) at an EC_50_ of 10 nM (means ± SEM, n = 4). (*G*, *H* and *I*) Lysolecithin-treated slice cultures were treated with 300 nM PIPE-307 and then immunostained using antibodies against MBP (myelin) or Caspr and Tuj1 (nodes of Ranvier). The stained MBP area was normalized to Hoechst count. Caspr puncta were normalized to Tuj1 stained area. MT7 was used as a positive control (means ± SEM, ANOVA with Tukey’s n = 4). D, Representative images of slices treated with vehicle alone, or lysolecithin with vehicle, 300 nM MT7, or 1 µM PIPE-307. (Scale bar: 25 µm.)

Next, we determined whether blockade of M1R in primary rat OPC cultures with PIPE-307 could promote OPC differentiation into oligodendrocytes. Upon blockade of M1R in OPCs with PIPE-307, we observed a concentration-dependent increase in the number of MBP^+^ oligodendrocytes with an EC_50_ of 38.6 nM, and efficacy comparable to that of T3 (triiodothyronine), a commonly used positive control (Fig. 4 *A* and *B*). Interestingly, we characterized a highly M3R-selective antagonist and found that it was not efficacious in this assay (*SI Appendix*, Fig. S4 *A* and *B*).

We next assessed whether time-limited application of PIPE-307 was sufficient to induce OPC differentiation. PIPE-307 was applied to OPCs for varying times, washed out, and cells allowed to continue differentiating for 72 h. Although no appreciable differentiation was observed with 2 h exposure, 8 h exposure was sufficient to elicit differentiation to an extent comparable to continuous 72 h exposure with either PIPE-307 or MT7. These suggest that M1R inhibition can act as a triggering event leading to differentiation and that chronic exposure is not needed to elicit a full effect (Fig. 4*C*). None of the previous anti-muscarinic compounds tested have influenced proliferation of OPCs in vitro or in vivo (8, 9). Similarly, we did not detect any change in the cell density of our purified OPC cultures after treatment with either PIPE-307 or MT7.

To test whether PIPE-307-induced oligodendrocytes were functionally competent (i.e., able to wrap axons), we employed a primary rat cortical myelination assay and measured average myelin segment lengths (MBP^+^/Tuj1^+^) (21–23). Continuous, straight segments were quantified and then averaged per oligodendrocyte to generate a myelination index. Using this assay, we observed a dose-dependent increase in myelination index (EC_50_ 16.2 nM, Fig. 4 *D* and *E*), showing that PIPE-307-induced oligodendrocytes are capable of wrapping axons. Induction was comparable to that observed using MT7.

### PIPE-307 Is Efficacious in Mouse Cortical Slice Cultures Following Acute Demyelination.

We next examined PIPE-307 in a more complex setting, i.e., mouse organotypic culture following lysolecithin-induced demyelination (23, 24). Brain slices were collected from P17 mice and demyelinated with lysolecithin. Slices were then treated with PIPE-307, harvested 3 d later, and *Mbp* mRNA assessed by quantitative PCR. Here, PIPE-307 dose dependently increased the expression of *Mbp* after lysolecithin challenge (EC_50_ 10 nM; Fig. 4*F*). To show the induction of myelinating oligodendrocytes after PIPE-307 treatment, we examined MBP expression 5 d after compound addition using an immunohistological endpoint and observed a significant increase in MBP levels (Fig. 4*G*). To show that axons were responding to remyelination by forming nodes of Ranvier, we quantified the number of Caspr puncta (contactin-associated protein). Upon myelination, nodal marker proteins like Caspr cluster in the axon (25). Given the relatively short duration of the experiment, each Caspr puncta was quantified independently to account for immature heminodes (26). A two-fold increase in Caspr puncta was observed in PIPE-307 treated slices compared to vehicle-treated slices (Fig. 4 *H* and *I*). These data show that in an acutely demyelinated ex vivo slice culture, OPCs respond to PIPE-307 and differentiate into MBP^+^ oligodendrocytes. Axons, in turn, respond to myelination by forming nodes of Ranvier as evidenced by Caspr staining.

### PIPE-307 Increases Oligodendrocytes in Human Cortical Slice Cultures.

**Fig. 5.**
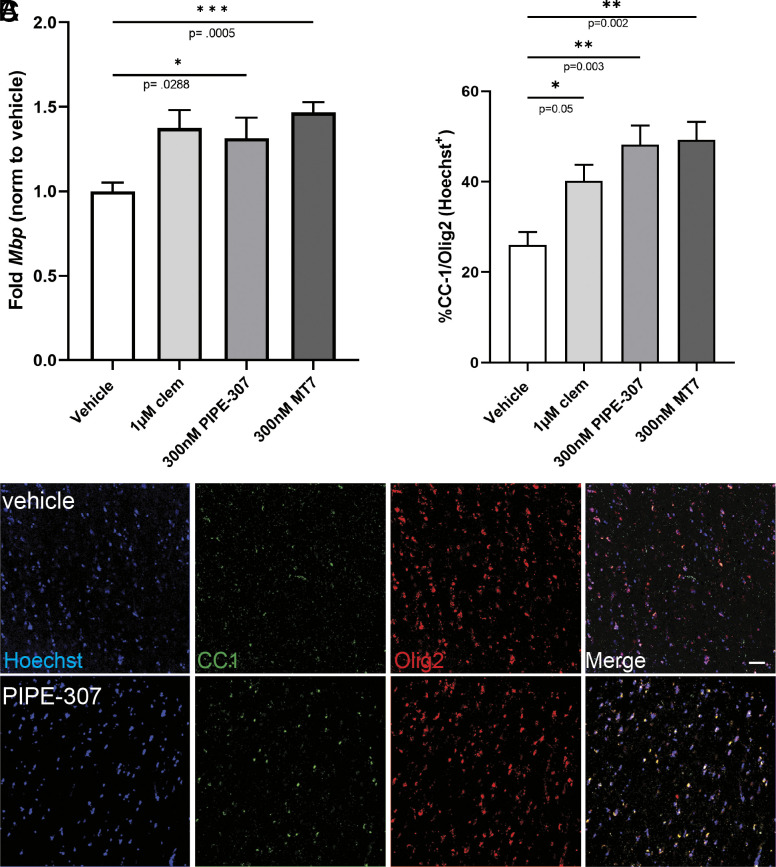
Treatment with PIPE-307 increases *Mbp* expression and the number of CC1^+^ oligodendrocytes in human cortical slice cultures. (*A*) Transcript analysis by qPCR of *Mbp* mRNA after treatment with 1 µM clemastine (clem), 300 nM PIPE-307, or 300 nM MT7 (means ± SEM, ANOVA with Dunnett’s, n ≥ 6 per group). (*B*) Quantitation of CC1^+^/Olig2^+^ oligodendrocytes following the same treatment as in A (means ± SEM ANOVA with Dunnett’s, n = 4 slices per group). (*C*) Representative immunohistochemistry of human organotypic cultures using antibodies against Olig2 (red), CC1 (green), and counterstained with Hoechst (blue). (Scale bar: 25 µm.)

To determine whether M1R antagonism could induce OPC differentiation in a human context, we tested PIPE-307 in human cortical slice cultures (27). Because lysolecithin-induced demyelination severely impacted slice health, only preexisting OPCs in a naive state could be tested. Slices were generated from adult donor cortical tissue and after 10 d in culture treated with PIPE-307, MT7, or clemastine. *Mbp* RNA was first evaluated by qPCR. Here, we observed a 1.31-fold induction in *Mbp* with 300 nM PIPE-307, a 1.38-fold induction with 1 µM clemastine, and a 1.47-fold induction with the selective M1R peptide antagonist, MT7 (Fig. 5*A*). We next used an immunohistochemical endpoint (CC1 expression) to quantify the number of oligodendrocytes. In this case, we observed a 1.85-fold increase with 300 nM PIPE-307, a 1.54-fold increase with 1 µM clemastine, and a 1.89-fold increase with MT7 (Fig. 5*B*). Only cells that colabeled with both Olig2 (oligodendroglial marker for OPCs and oligodendrocytes) and Hoechst were quantified (Fig. 5*C*).

These data show that treatment with selective M1R antagonists (PIPE-307 or MT7) increases the number of oligodendrocytes in a human context. Moreover, they show that PIPE-307 has the capacity to induce differentiation of aged OPCs as the donor tissue obtained for this experiment came from an individual >50 y of age. Of note, the M3R selective small-molecule antagonist was not active in this context highlighting the importance of blocking M1R over M3R in an adult human setting (*SI Appendix*, Fig. S4*C*).

### PIPE-307 M1R Occupancy in the Brain.

**Fig. 6.**
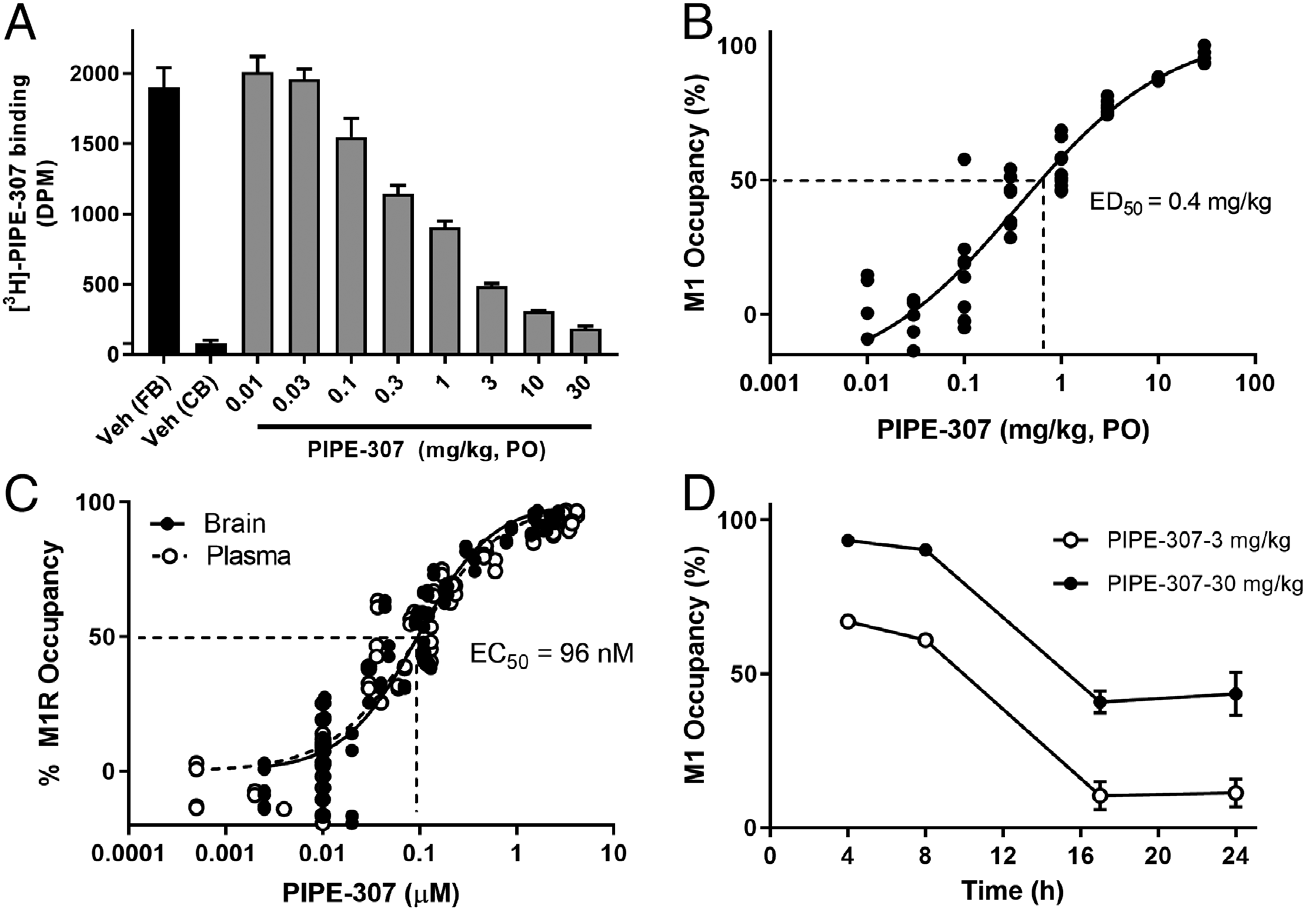
In vivo receptor occupancy profile of PIPE-307. (*A*) Oral dosing to mouse results in dose-dependent inhibition of total [3H]-PIPE-307 binding in mouse brain (means ± SEM, n ≥ 4/group). The forebrain (FB) was used to determine total binding, a region with high M1 receptor expression. Nonspecific binding was determined using the cerebellum (CB), a region low in M1 receptor expression. (*B*) Represents the % M1 receptor occupancy of individual subjects plotted as a function dose. PIPE-307 shows a dose-dependent effect with an ED_50_ = 0.4 mg/kg. (*C*) Represents the receptor occupancy of individual subjects plotted as a function of plasma and brain PIPE-307 concentrations. The estimated plasma and brain EC_50s_ were similar at 96 nM. The resulting brain to plasma ratio = 1. (*D*) Time course data following a single oral dose of 3 mg/kg and 30 mg/kg, plotted as % occupancy (as means ± SEM, n ≥ 4/group). 30 mg/kg, PO displays rapid and full occupancy at early time points and falls to ~50% occupancy by 16 h; 3 mg/kg achieves ~50% occupancy for about 10 h postdose. %occupancy = 100 [(treatment specific binding/total baseline specific binding) * 100].

To ensure the selection of a proper dose that would provide adequate in vivo coverage for subsequent efficacy experiments, we measured mouse M1R occupancy of PIPE-307 in the CNS. Having shown PIPE-307’s selectivity for M1R in vitro, competition experiments were performed using [^3^H]-PIPE-307 as a radiotracer in vivo. PIPE-307 was dosed orally 2 h prior to radiotracer injection (IV bolus) and brains were harvested 5 min later. A dose-dependence curve was generated using PIPE-307 at doses ranging from 0.01 to 30 mg/kg. We observed that a single oral dose of PIPE-307 inhibited M1R-selective radioligand binding to the mouse forebrain in a dose-dependent manner (ED_50_ of 0.4 mg/kg, Fig. 6 *A* and *B*). The resulting total plasma and brain EC_50_s were both determined to be 96 nM, which is consistent with a brain-to-plasma ratio (or K_p_) of 1 (Fig. 6*C*). Correcting for protein binding (91.2% mouse plasma protein binding and 93.2% mouse brain tissue binding), the unbound plasma EC_50_ for PIPE-307 was calculated to be 9 nM, and the unbound brain EC_50_ was calculated to be 7 nM. The unbound brain-to-plasma ratio (or K_puu_) was therefore determined to be 1.3. These results demonstrate that PIPE-307 distributes effectively into the brain following oral dosing.

Time course studies were then performed using 3 and 30 mg/kg doses with the brains harvested 2 to 24 h postdose. At 3 mg/kg, ≥60% M1R occupancy was achieved for at least 8 h at which time the unbound brain concentration was determined to be 8 nM. By 17 h, this had declined to ≤10% occupancy and the drug level of PIPE-307 in the brain was below the limit of quantification. By extrapolation, over 50% occupancy was maintained for approximately 10 h following an oral dose of 3 mg/kg. At the 30 mg/kg dose, greater than 90% occupancy of the M1 receptor was maintained for at least 8 h (unbound brain concentration of PIPE-307 = 116 nM). By 17 h, the M1R occupancy had declined to 40% (unbound brain concentration of PIPE-307 = 6 nM) and this level was maintained through 24 h. By extrapolation, ≥50% occupancy was maintained for approximately 16 h (Fig. 6*D*). In contrast, at a dose of 10 mg/kg, clemastine occupied only 46% of the M1 receptor at 1 h, and rapidly declined to 20% by 4 h (*SI Appendix*, Fig. S5).

### PIPE-307 Is Efficacious in the Mouse MOG-EAE Model of Demyelination.

Given these occupancy values, we proceeded to evaluate PIPE-307 in an in vivo inflammatory demyelination model, MOG-EAE (myelin oligodendrocyte glycoprotein-induced experimental autoimmune encephalitis). Our in vitro data in OPCs demonstrated that 8 h application of PIPE-307 was sufficient to elicit OPC differentiation. Therefore, following immunization with MOG, mice were dosed orally once daily for 22 d with vehicle or PIPE-307 (3 or 30 mg/kg) starting on day 0.

**Fig. 7.**
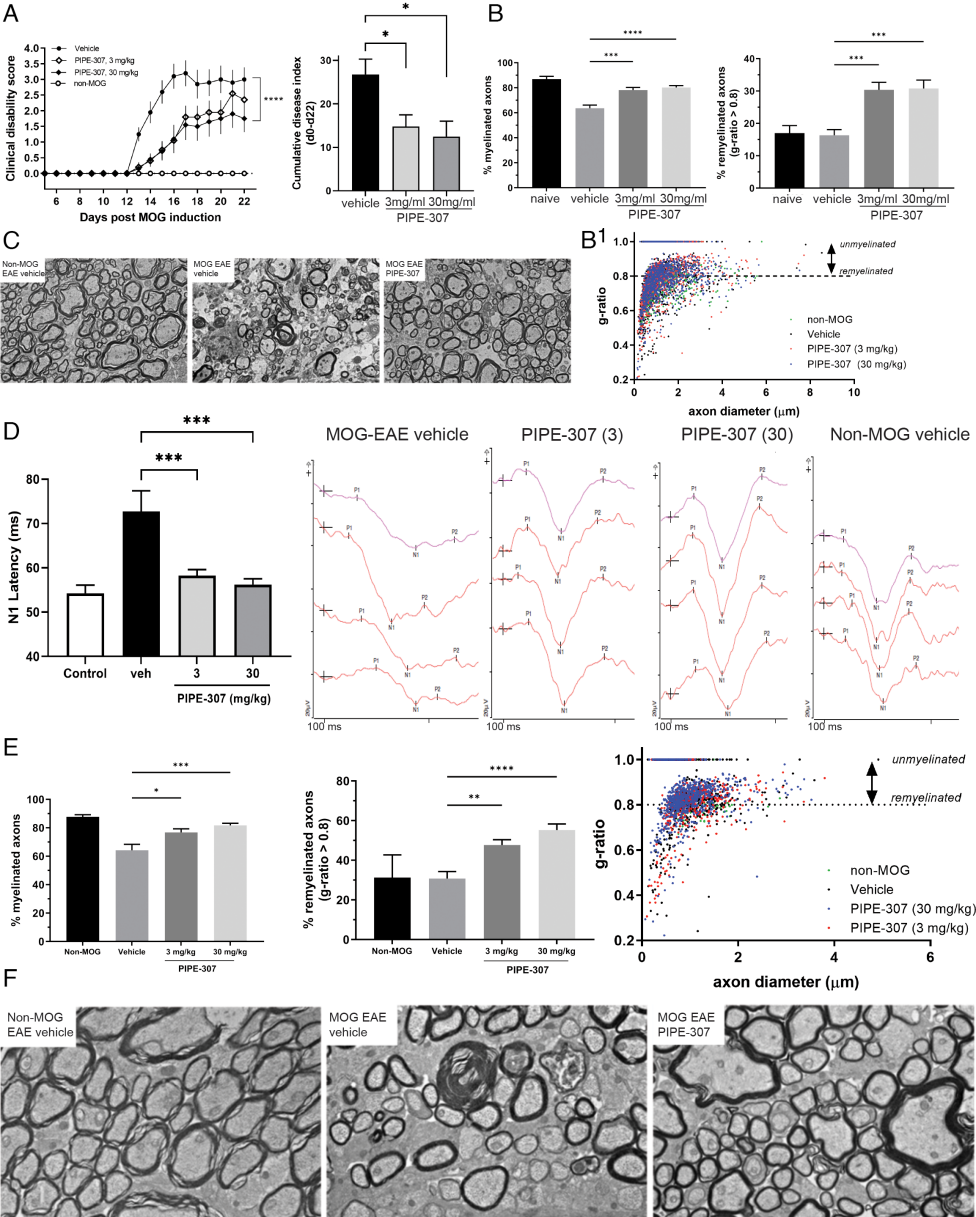
PIPE-307 improves clinical score and induces remyelination in the mouse EAE model. (*A*), (*Left*) Daily treatment of PIPE-307 from day 0 decreases the clinical severity in the MOG-EAE model; (*Right*) cumulative disease index, where scores from day 0 to 22 were summed (means ± SEM; ANOVA with Tukey’s, n = 10 for all groups, ****P < 0.0001, *P < 0.05). (*B*). PIPE-307 increases the number of myelinated and remyelinated axons in the lumbar spinal cord as defined by a g-ratio > 0.8, but less than 1. (*B*), *left,* Bar graph of % total myelinated axons; (*B*), *right*, bar graph of % remyelinated axons (means ± SEM, ANOVA with Tukey’s, n = 4 for non-MOG group, n = 10 for all other groups, ***P < 0.001); (*B*^1^) scatterplot of g-ratios as a function of axon diameter. (*C*) Representative electron micrographs of spinal cord tracts from non-MOG-EAE vehicle, MOG-EAE vehicle, and MOG-EAE PIPE-307 (30 mg/kg) treated mice. (*D*) Treatment with PIPE-307 improves visually evoked potential N1 latency times in the optic nerve (means ± SEM, ANOVA with Dunnett’s post hoc test, n = 16 to 20, ***P < 0.001). *Right*, Representative traces. (*E*) PIPE-307 increases the number of total myelinated and remyelinated axons in the optic nerve tract. *Left*, Bar graph of % total myelinated axons; *right*, bar graph of remyelinated axons (means ± SEM, ANOVA with Dunnett’s post hoc test, n = 3 for non-MOG, n = 13 to 17, ***P < 0.001); *right*, scatterplot of g-ratios as a function of axon diameter (means ± SEM, ANOVA, Tukey’s post hoc, n = 3 for non-MOG group, n = 13 to 17 for remaining groups, **P < 0.01, ****P < 0.0001). (*F*) Representative electron micrographs of optic nerve tracts from non-MOG-EAE vehicle, MOG-EAE vehicle, and MOG-EAE PIPE-307 (30 mg/kg) treated mice.

Clinical disability was first observed on day 13 and reached a mean peak score of 3.2 on day 17. In MOG-induced animals treated with 3 mg/kg PIPE-307, the mean clinical score increased with time but was blunted relative to vehicle controls. At 30 mg/kg, clinical scores were reduced further and while significant to vehicle, were not significantly different from the 3 mg/kg group (Fig. 7*A*). A cumulative disease index was generated by summation of the clinical scores across the study. Comparison in this manner showed significant improvement in both 3 and 30 mg/kg PIPE-307-treated groups versus vehicle.

Since the most profound demyelination is known to occur at the lumbar spinal cord, this region was analyzed by electron microscopy and *g*-ratios were measured as a direct indicator of myelination status (9). In non-MOG-immunized mice, 86.9% of axons measured were myelinated (*g*-ratio < 1), whereas in MOG-immunized vehicle-treated mice, only 63.6% of the axons were myelinated. It is generally thought that thinly myelinated axons with a g-ratio > 0.8 represent the population of remyelinated axons, whereas axons with a lower g-ratio are thought to be surrounded by preexisting myelin (9). While this analysis includes a large distribution of axons, significant changes in the population of g-ratios are suggestive of remyelination. At 3 mg/kg PIPE-307, 78.1% of the axons were myelinated, and 80.2% were myelinated at a treatment dose of 30 mg/kg (Fig. 7*B*). Further analysis of remyelinated axons (those with a g-ratio > 0.8) showed that there were significantly more remyelinated axons after 3 or 30 mg/kg PIPE-307 (9). Whereas MOG-immunized mice 16.3% remyelination, with PIPE-307, this percentage was significantly higher, namely 30.4% with 3 mg/kg, and 30.8% with 30 mg/kg (Fig. 7*B*). To determine whether we could ascertain any axonal protection, we attempted to analyze the total axon counts from our EM samples. Unfortunately, at the time point analyzed, debris and distressed axons with dark axoplasm made it difficult to identify and quantify the population of unmyelinated axons accurately.

The optic nerve is a heavily myelinated tract that is sensitive and uniquely amenable to monitoring demyelination. Visual evoked potential (VEP) latency is a noninvasive, clinically translatable method of measuring myelination and conduction velocity in the optic nerve (28, 29). VEP latencies from the same mice as described above were recorded on nominal day 21 (day 21 to 24). Control non-MOG-immunized mice had a mean N1 latency time of 54.3 ms, whereas latency time was delayed in MOG-immunized animals to 72.7 ms. PIPE-307 treatment significantly reduced N1 latency (increased conduction velocity) suggesting remyelination. Specifically, the 3 mg/kg treatment group showed a 14.4 ms improvement (to 58.3 ms), and the 30 mg/kg treatment group showed a 16.5 ms improvement (to 56.2 ms) (Fig. 7*D*). *g*-ratios in the optic nerve were also determined. In non-MOG-immunized mice, 87.7% of the axons were myelinated (*g*-ratio < 1). In the optic nerves of MOG-immunized, vehicle control mice, 61.5% of the axons were myelinated. With PIPE-307 treatment, significantly more myelinated axons were observed, with 76.8% myelinated at 3 mg/kg and 81.7% at 30 mg/kg (Fig. 7*E*). PIPE-307 also significantly increased the number of remyelinated axons (g-ratio > 0.8) with 47.7% at 3 mg/kg and 55.2% at 30 mg/kg. In contrast, only 30.7% of axons were remyelinated in vehicle-treated MOG-immunized mice (Fig. 7*E*).

### PIPE-307 in the Rat Novel Object Recognition Test.

Given the well-known effects of anti-muscarinics (e.g., scopolamine) in tests of cognition, we wanted to determine whether selective M1R blockade would produce similar effects using a rat novel object recognition test (NOR) (30, 31) PIPE-307 was evaluated 2 h after a single oral dose of 1, 3, or 30 mg/kg. These dose levels were selected based on rat receptor occupancy studies where approximately 50%, 75%, and 100% M1R blockade in the CNS were observed, respectively (*SI Appendix*, Fig. S6). Scopolamine (0.3 mg/kg, dosed subcutaneously) was used as positive control. As expected, a significant reduction in recognition index was observed in the scopolamine group. PIPE-307, on the other hand, at 1 and 3 mg/kg did not produce any discernable decrease in the recognition index. At 30 mg/kg, a reduction was observed, however, this effect did not reach statistical significance. Furthermore, a group tested 24 h after 30 mg/kg dosing, at which time the CNS M1R occupancy in rats is below 10%, showed a recognition index comparable to vehicle (*SI Appendix*, Fig. S7).

## Discussion

Identifying novel approaches to limit or prevent axonal degeneration associated with chronic progressive MS is of critical importance. Current therapies targeting the immune system do not address this unmet medical need. Mounting evidence suggests that the myelin sheath is indispensable to axonal survival by providing physical as well as metabolic support (32). Previous work by Mei et al., provided clear evidence using the EAE mouse model that remyelination was sufficient to preserve axonal integrity and neuronal function under inflammatory demyelinating conditions. Further, genetic approaches validated M1R as a therapeutic target for remyelination. In this present study, we disclose PIPE-307, a potent and selective M1R antagonist, and highlight its therapeutic potential as an oral treatment for MS through the promotion of remyelination. PIPE-307 was the product of a focused medicinal chemistry effort directed at identifying an orally bioavailable M1R selective antagonist that readily crosses the blood–brain barrier and occupies central M1 receptors. Our internal efforts focused on improving the selectivity for M1R versus the other muscarinic receptors and improvement of CNS drug-like properties in contrast to nonselective antagonists such as clemastine or benztropine.

Using multiple endpoints, we demonstrate that antagonizing M1R with PIPE-307 is effective in promoting OPC differentiation into oligodendrocytes. Using in vitro rodent assays, PIPE-307 was efficacious in differentiating rodent OPCs into MBP^+^ oligodendrocytes in dissociated cultures. In acutely demyelinated mouse brain slice cultures, we observed an increase in both myelin basic protein mRNA and protein, as well as an increase in nodes of Ranvier (as assessed by Caspr) in organotypic brain slice cultures, confirming functional remyelination in a complex system. Our in vitro data also suggest that abnormal levels of acetylcholine, for example, in a diseased state, could limit OPC differentiation into oligodendrocytes (33).

We show that PIPE-307 is efficacious in increasing the number of CC1^+^ oligodendrocytes in a human brain slice culture. These results confirm the ability of modulating the muscarinic pathway to increase differentiation of aged OPCs. These findings are consistent with the study by Wang et al., demonstrating that clemastine, as a single agent, can induce OPC differentiation in aged mice (34). Isolated OPCs from aged mice, on the other hand, appear to lose the capacity to respond to anti-muscarinics in the absence of metabolic manipulation (35). While unclear what accounts for these discrepancies, these could be explained by differences in experimental contexts. We would also emphasize that the effects of PIPE-307 were comparable to the highly potent and selective M1R peptide antagonist, MT7, thus highlighting the role of M1R in driving these effects. Interestingly, application of an M3R selective small molecule (Banyu, Compound 51a) did not induce OPC differentiation in our rat OPC or in human slice cultures (36). While we cannot rule out the possible contribution of M3R in conjunction with other prodifferentiation mechanisms, in the contexts we tested, we did not observe stand-alone efficacy with an M3R-selective antagonist and must conclude that M1R is the dominant negative regulator of oligodendrocyte differentiation and myelination.

Oral delivery of PIPE-307 to mice resulted in increased remyelination in the MOG-EAE model of inflammatory demyelination and functional recovery. These effects were observed at dose levels where M1R is selectively antagonized in the CNS longer and to a greater extent than what could be achieved with clemastine, whose mechanism of action on OPCs was shown to be via M1R (8, 9). Both 3 and 30 mg/kg doses of PIPE-307 resulted in comparable effects on remyelination as assessed by VEP (a functional endpoint for evaluating myelination status) and myelin g-ratios in both spinal cord and optic nerve. Consistent with the study by Mei et al., which showed that heterozygous knockout of M1R in OPCs conferred comparable protection to the homozygous knockout mice in the MOG-EAE model of MS, our data would suggest that full M1R occupancy is not needed to achieve full efficacy.

Our data also suggest that pulsed dosing may be sufficient for remyelination. Namely, in vitro data in OPCs demonstrate that 8 h exposure to PIPE-307 is sufficient to trigger differentiation, with efficacy comparable to continuous application of either PIPE-307 or MT7. More importantly, significant efficacy was also achieved in the MOG-EAE setting at a daily oral dose of 3mpk PIPE-307, a dose where 10 h pose dosing, the occupancy of the M1R in the CNS will drop below 50%. Although there was trending efficacy in the clemastine ReBuild trial, only limited M1R occupancy was achieved (37). While full M1R occupancy may not be required for OPC differentiation and remyelination, a clinical trial with PIPE-307 would allow for M1R occupancy to be tested beyond what was achieved by clemastine in an M1R-selective manner. Together, these would allow for a complete, targeted evaluation of M1R antagonism for remyelination.

Anti-muscarinic compounds have generally been viewed as having a negative impact on cognition (38). The contribution of M1R to these side effects has been an area of significant research. M1R KO mice have been extensively evaluated and do provide evidence that this receptor is involved in many aspects of learning and memory (38–41). It is interesting to note, however, that not all reports observe a negative impact of M1R KO on cognitive performance (42). Furthermore, scopolamine has been reported to produce comparable cognitive deficits in WT and M1R KO mice in the Morris water maze test (38). Observations made using scopolamine or other nonselective antimuscarinics should not, therefore, be interpreted as mediated solely by M1R. Using the novel object recognition test, we evaluated PIPE-307 at dose levels that achieve near-complete M1R occupancy in the CNS. PIPE-307 had no impact on NOR at 1 and 3 mg/kg, and showed a modest, variable, response at 30 mg/kg 2 h postdose. This is in stark contrast to scopolamine and other nonselective anti-muscarinics and suggests that dose levels capable of promoting remyelination should not carry significant cognitive liability.

In a completed Phase I healthy volunteer study with PIPE-307, we assessed measures typically impacted by scopolamine, including psychomotor function, attention, memory, and executive function at key PK time points during the SAD and MAD (single and multiple ascending dose) cohorts. At doses equivalent to nearly 80% occupancy of M1R in the brain, we observed no evidence of a negative effect on higher cognitive function in human subjects (https://clinicaltrials.gov/study/NCT04725175). PIPE-307 is not intended as a stand-alone treatment for relapsing-remitting MS. It is intended to be used in conjunction with immunomodulatory disease-modifying therapies—as it is essential to prevent relapses and new damage while also engendering repair of previous areas of demyelination. Since the ReBuild trial in chronic optic neuropathy using VEPs as a primary endpoint was successful, our approach with PIPE-307 would be similar in design with a greater magnitude of effect anticipated given greater coverage of the target receptor at acceptable doses (38).

Together, these data validate PIPE 307 as a brain-penetrant M1R-selective antagonist with drug-like properties, for use in demyelinating diseases such as MS. Specifically, these data confirm previous findings that M1R acts as a brake on OPC differentiation and remyelination in vitro and in vivo. Of note, the effects of M1R blockade observed with PIPE-307 are consistent with previous genetic M1R knockout data (9). The effectiveness of PIPE-307 in the in vivo and in vitro preclinical models lend support for M1R as a target for remyelination and underscore the potential use of PIPE-307 for treating MS and other demyelinating diseases.

## Materials and Methods

### [^3^H]-NMS Human M1R Membrane Binding.

hM1-5 membranes were obtained from Perkin Elmer (Waltham, MA). Test compound was serially diluted and added to 0.158 nM [3H]-NMS (Perkin Elmer, Waltham, MA) and 20 µg of membrane diluted in assay buffer (50 mM Tris, 154 mM NaCl, and 0.1% Tween-20, pH 7.4). Plates were shaken @ 300 rpm for 2 h at room temperature. Membranes were filtered on PEI-coated UniFilter-96 GF/B, White 96-well Barex Microplates (Perkin Elmer, Waltham, MA) on a Brandel harvester (Gaithersburg, MD). The plates were washed twice (50 mM Tris and 154 mM, NaCl, pH 7.4) and allowed to dry at room temperature. Once dry, plate bottoms were sealed, 50 µL of Betaplate Scintillation cocktail (Perkin Elmer, Waltham MA) was added to each well, and the plate tops were sealed. Plates were read on the TopCount (Perkin Elmer, Waltham, MA).

### [^3^H]-PIPE-307 Mouse and Human Brain Homogenate Binding.

For mouse binding, forebrain tissue was obtained from adult M1R knockout animals on a C57BL/6 background or wild-type littermate controls (Jonah Chan lab, UCSF). For human binding, cortical tissue was obtained from two adult male Caucasian donors (Cureline, Brisbane CA). The human samples were deidentified prior to use. For mouse and human binding, fresh frozen tissue was thawed and homogenized with a glass Dounce tissue homogenizer in ice-cold buffer (50 mM Tris-HCl, 154 mM NaCl, pH 7.4). Homogenized samples were centrifuged (20,000 g, 10 m). Pellets were resuspended in buffer at 100 mg/mL, −80 °C until use.

[^3^H]-PIPE-307 (Vitrax, Placentia, CA) was tested at a concentration range of 100 pM–10.0 µM to determine B_max_ and used at a final concentration of 3 nM for competition binding. Radioligand was diluted in assay buffer. [3H]-PIPE-307, 1 mg/mL membranes and PIPE-307 (for competition binding) were added to a deep-well plate (Thermo Fisher, Waltham, MA). Plates were shaken at 300 rpm for 2 h at room temperature. Membranes were filtered on PEI-coated UniFilter-96 GF/B, White 96-well Barex Microplates (Perkin Elmer, Waltham, MA) on a Brandel harvester (Gaithersburg, MD). The plates were washed twice (50 mM Tris, 154 mM, NaCl, pH 7.4) and allowed to dry at room temperature. Once dry, plate bottoms were sealed, 50 µL of Betaplate Scint cocktail (Perkin Elmer, Waltham MA) added to each well and the plate tops were sealed. Plates were read on the TopCount (Perkin Elmer, Waltham, MA).

### Calcium Mobilization in Human Cells Overexpressing mAChR.

CHO-K1 cells stably expressing human M1–M5 muscarinic receptor with aequorin (Perkin Elmer, Waltham, MA) were grown in Ham’s F-12 media (Invitrogen, Carlsbad, CA), 10% FBS (ATCC, Manassas, VA) with 0.4 mg/mL geneticin and 0.25 mg/mL Zeocin (Invitrogen, Carlsbad, CA) or for hM5 cells, Ham’s F-12, 10% FBS, 0.4 mg/mL geneticin, 5 µg/mL puromycin (Invitrogen, Carlsbad, CA). Cells were lifted with Accutase (Sigma, St Louis, MO), pelleted and resuspended in assay buffer (DMEM/F-12 with HEPES (Invitrogen, Carlsbad, CA) +0.1% protease-free BSA (Fisher Scientific, Waltham, MA) and coelenterazine h (Promega, Madison WI) at a final concentration of 5 µM, and incubated for 4 h at room temperature in the dark. Cells were then plated at 5 × 10^5^ cells/well in 96-well white-walled, tissue culture-treated, clear-bottom plates (VWR, Radnor, PA). Compounds were added and incubated for 30 min. Calcium mobilization was measured using the FlexStation 3 (Molecular Devices, San Jose, CA). EC_80_ ACh (M1R 60 nM, M2R 2 µM, M3R 60 nM, M4R 60 nM, and M5R 125 nM) was pipetted, and the increase in luminescence was measured over time. Antagonist activity was analyzed as a concentration-dependent inhibition of in the EC_80_ ACh-induced release.

## Supplementary Material

Appendix 01 (PDF)

## Data Availability

All study data are included in the article and/or *SI Appendix*.
