# Supplementary material for: Targeting the muscarinic M1 receptor with a selective, brain-penetrant antagonist to promote remyelination in multiple sclerosis
Source: Proc Natl Acad Sci U S A. 2024 Jul 31;121(32):e2407974121. doi: 10.1073/pnas.2407974121 (PMC11317586; doi:10.1073/pnas.2407974121)
Supplement: Supplementary file 1 — Appendix 01 (PDF) [file pnas.2407974121.sapp.pdf]

Supplementary Figure 1.

A.

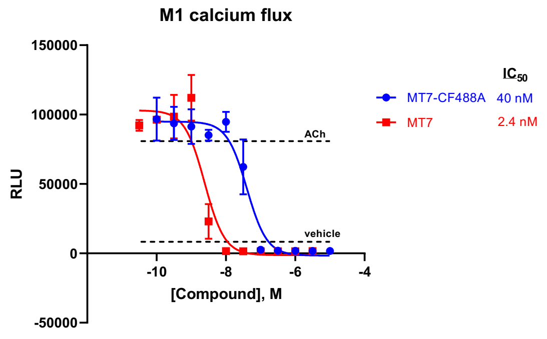

B.

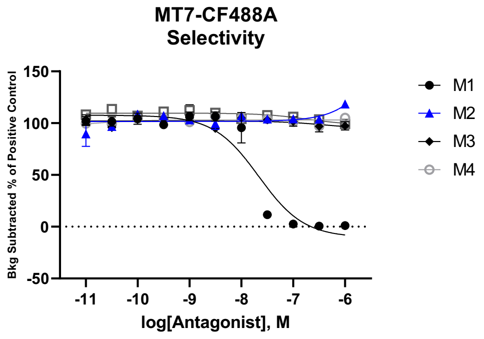

C.

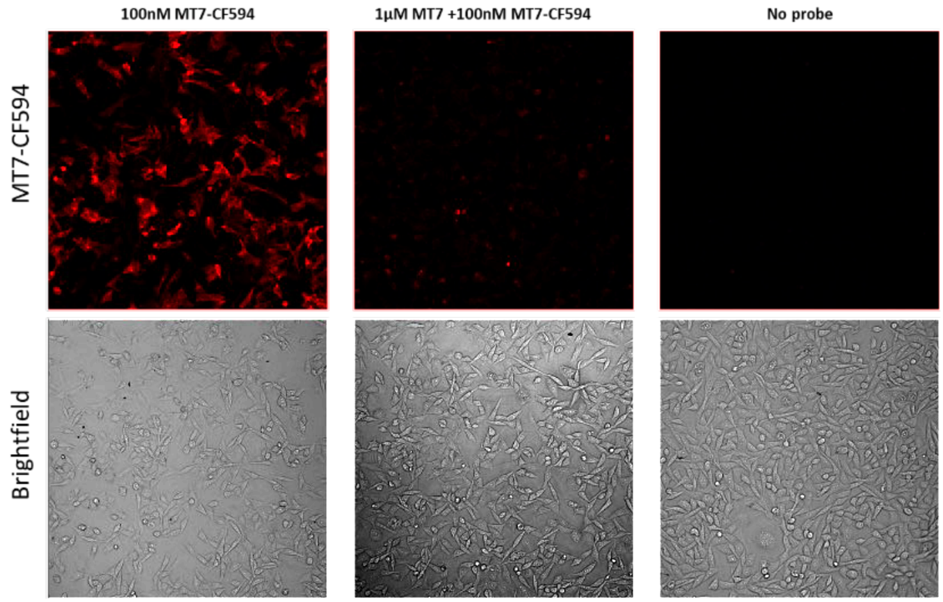

Supplementary Figure 1. MT7-CF488 probe validation. A. MT7CF-488A evaluated in calcium mobilization assay in M1R overexpressing CHO-K1 cells, IC_{50} 40 nM. B. MT7-CF488A retains functional selectivity against M1R in calcium mobilization assay. C. Characterization of MT7-CF594 probe. M1R overexpressing CHO-K1 cells were incubated with 100 nM MT7-594 (left) or co-incubated with 1μM MT7 (right). Co-incubation with MT7 results in near complete loss of fluorescent signal, comparable to no probe control (right).

Supplementary Figure 2.

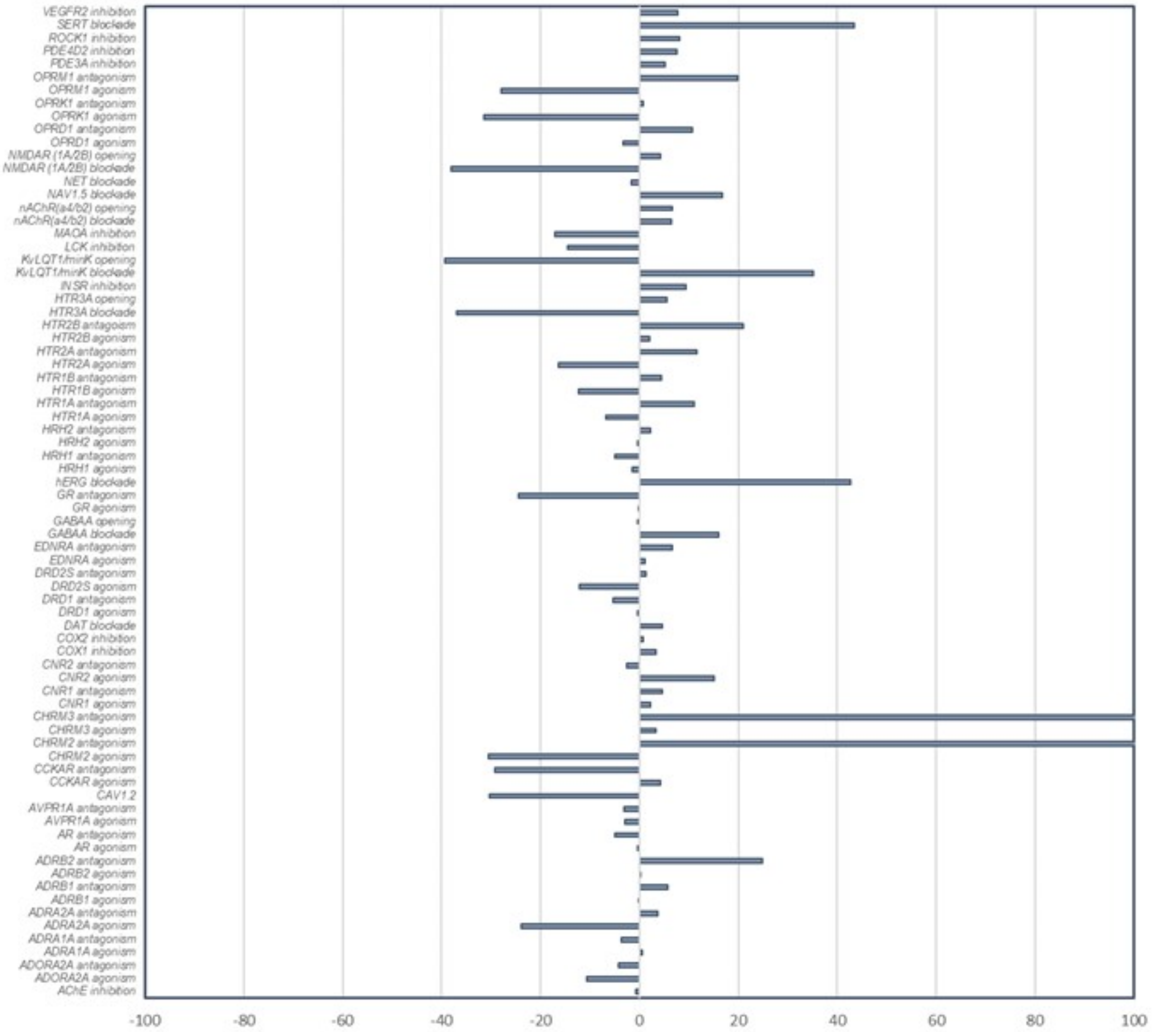

Supplementary Figure 2. Results from Eurofins Safety47 panel against a variety of receptors. PIPE-307 was tested at a concentration of 10 µM and was below 50% activity for all non-muscarinic receptors tested.

Supplementary Figure 3.

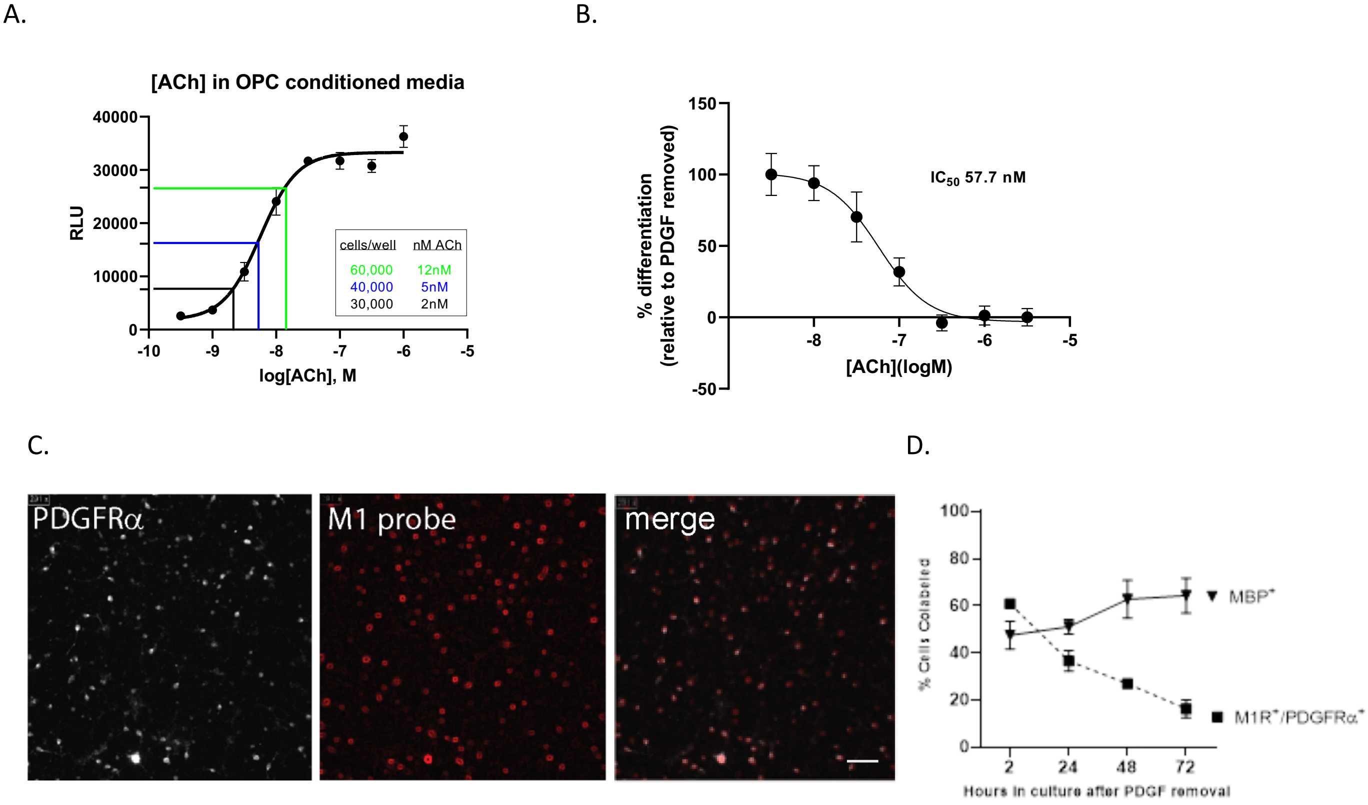

Supplementary Figure 3. OPC cultures used in this paper (60,000 cells/well) contain 12 nM acetylcholine. A. Acetylcholine concentrations were measured in OPC conditioned media from wells containing different densities of OPCs using a standard curve generated using known concentrations of acetylcholine (means ± SEM, n=3/data point. B. ACh suppresses OPC differentiation after PDGF withdrawal. Rat OPCs were plated in $PDGF^+$ media. PDGF was removed and OPCs were cultured in varying concentrations of acetylcholine. Cells were stained with MBP antibody and counted. 100% was set to differentiation in the absence of acetylcholine. (means ± SEM, n=15 cells/ data point). C. $M1R^+$ OPCs diminish over the course of differentiation. OPCs express M1R. 72 h after plating, cultured rat OPC cultures were stained with an OPC antibody marker (PDGFRα, white) and the M1 probe (MT7-CF594, red). Scale bar:25 µm. D. Upon PDGF removal, OPCs differentiate into oligodendrocytes. Over the course of differentiation, $M1R^+$/$PDGFR\alpha^+$ OPCs decrease with a concomitant increase in MBP^+ oligodendrocytes (means ± SEM, n ≥ 13 wells/data point).

Supplementary Figure 4.

A.

Human receptor	[^{3}H]NMS membrane binding
**M1**	4670 ×/÷ 1.00
**M2**	6730 ×/÷ 1.00
**M3**	25.5 ×/÷ 1.05
**M4**	3600 ×/÷ 1.00
**M5**	Not determined

B.

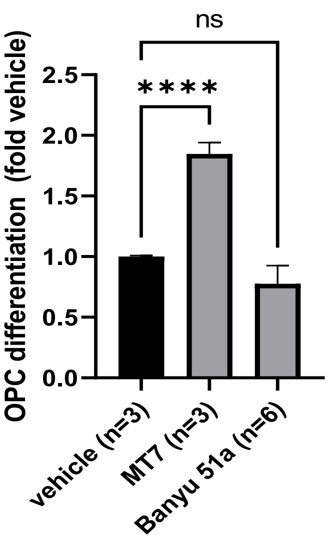

C.

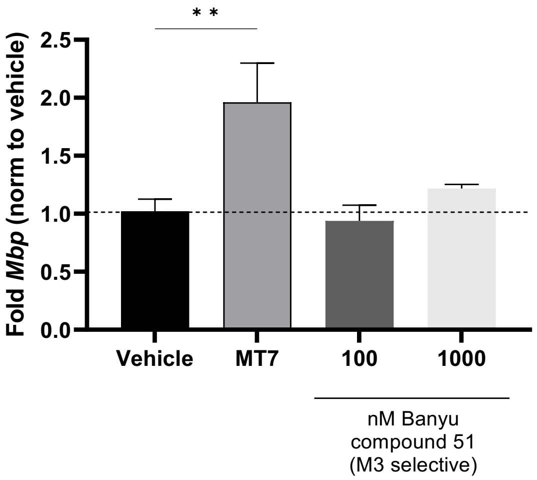

Supplementary Figure 4. Small molecule M3R selective antagonist (based on Sagara et al., 2006, Compound 51a) does not show activity in rat OPC or human slice culture assays. The molecule was synthesized and tested in a [3H]-NMS binding assay using membranes overexpressing various human muscarinic receptor isoforms. K_is expressed as geometric mean, n=2. B. The M3R selective antagonist was assayed in the rat OPC differentiation assay. At the highest concentration tested (3 µM) no significant impact on OPC differentiation was observed. 300nM MT7 (M1 selective) displayed significant activity relative to vehicle (means ± SEM, ANOVA with Tukey's, **** p <0.0001). C. Human slice cultures were treated with compound 51a and *Mbp* RNA analyzed by qPCR. No significant induction of *Mbp* RNA was observed at 100 or 1000 nM. MT7 was used a positive control (means ± SEM, n ≥ 3. ** p > 0.01).

Supplementary Figure 5.

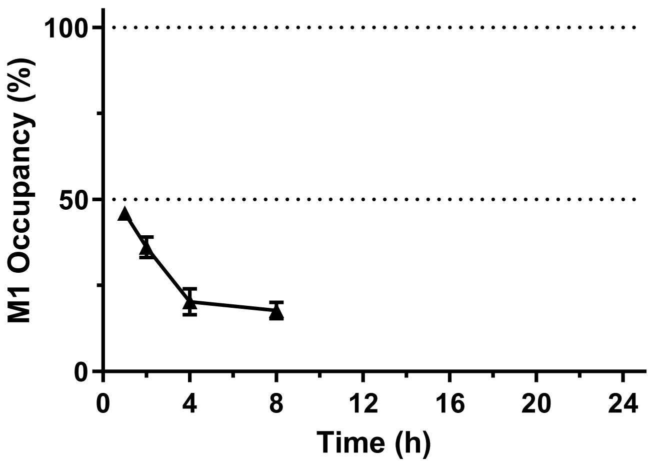

Supplementary Figure 5. Clemastine occupancy of M1R. Clemastine (10 mg/kg) achieves 46% occupancy at 1 hour post-dose, then rapidly declines to 36% at 2 hours, and 18% by 8 hours (means ± SEM).

Supplementary Figure 6.

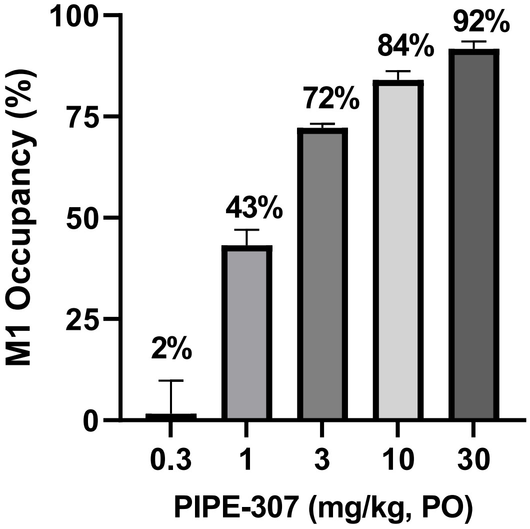

Supplementary Figure 6. *In vivo* receptor occupancy profile of PIPE-307 in rat. Oral dosing to results in a dose dependent increase in M1 receptor occupancy (means ± SEM, 6 animals per group). %occupancy = 100((treatment specific binding/total baseline specific binding) *100).

Supplementary Figure 7.

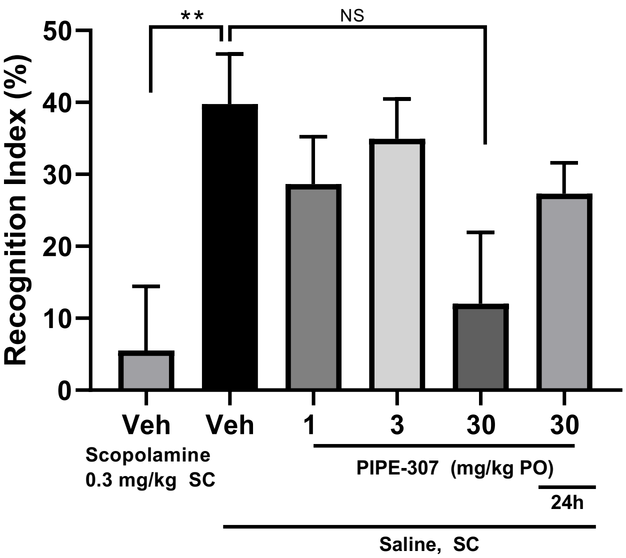

Supplementary Figure 7. Scopolamine at 0.3 mg/kg served as a positive control and resulted in a significant impairment of recognition index as compared to vehicle/saline controls (means ± SEM, ANOVA with Tukey's, n≥12, ** p<0.01,). 2h post, 30 mg/kg PIPE-307 reduced the recognition index but was not statistically different from vehicle/saline. The impairment of recognition index was normalized at 24h post-PIPE-307, suggesting that the modest effect of PIPE-307 on object recognition was transient.

Table 1

Properties	*In vitro* Profile				
Receptor	hM1	hM2	hM3	hM4	hM5
Radioligand Binding K_i (nM)	4.6	310	77	52	79
Fold selectivity	-	67x	17x	11x	17x
Functional Ca^{+2} mobilization IC_{50} (nM)	3.8	1600	210	110	3600
Fold selectivity	-	420x	55x	29x	950x

Table 1. Summary table of PIPE-307 i*n vitro* selectivity profile in [^{3}H]-NMS radioligand binding and calcium mobilization assays across the various human muscarinic isoforms.

Table 2

	Human		Mouse	
		Wildtype	Heterozygous	Homozygous
B_{max}	348	1971	1025	33
K_d (nM)	6.3	5.4	11.1	NA
K_i (nM)	1.5	1.2	NA	>10000

Table 2. Summary table of [^{3}H]-PIPE-307 radioligand binding using human versus mouse brain tissue homogenates. NA = not applicable.

Supplementary Materials and Methods

M1R labelling with MT7 probe and immunohistochemistry

Fresh frozen human brain tissue sections were obtained from Biochain Institute, IC (Novato, CA). Fresh frozen MS donor brain tissue was provided and characterized by the Netherlands Brain Bank. Sections were incubated with MT7-CF488A at 1µM diluted in HBSS with 0.1% BSA for 1 hour, rinsed briefly, then fixed with 4% PFA for 15 minutes. Sections were washed 3 times with DPBS. Antibody were diluted at 1:250 in DPBS containing 10% normal goat serum and 0.25% Triton-X 100 and applied overnight at 4°C. Antibodies used were mouse NG2 (Millipore, AB5320) and rabbit Olig2 (Cell Marque, Rockland, CA). For rat tissue, PDGFRα antibody (Cell Signaling Technologies, Danvers, MA), Sections were washed 3x in DPBS then secondary antibodies diluted in DPBS at 1:250 containing 1:2000 Hoechst 33342 were applied (Invitrogen, Carlsbad, CA). Sudan Black was used to stain for myelin in human sections (Biotium, Fremont, CA). Secondary antibodies used were all from Invitrogen (Carlsbad, CA), goat anti-rabbit Alexa594 and goat anti-mouse Alexa647.

MT7 probe evaluation

Calcium mobilization was performed as described in main text using CHO-K1 cells overexpressing M1, 2, 3, or 4 receptors. Fluorescent microscopy was also performed with these cells. Cells were plated on a Viewplate (Perkin Elmer, Waltham, MA). After adhesion, cells were incubated in culture media containing 100 pM or 100 nM of MT7-CF488A for 24 hours, washed three times with PBS and imaged. For native protein gels, 8µg membranes from hM1R CHO-K1 cells, 15µg P19 mouse forebrain homogenate (homogenized in PBS only), 15µg M1R KO brain homogenate, 15µg WT brain homogenate, or 8µg CHO-M1 membranes (50µg/50µL) were incubated MT7-CF488A for 30m at room temperature. 5x native sample loading buffer was added, then samples were run on a Novex 4-20% Tris-Glycine gel (Invitrogen, Carlsbad, CA). Gels were transferred to PVDF and fluorescence imaged on a Storm 825 scanner (Cytiva, Marlborough, MA). Presumably because of the small molecular weight, free non-M1R bound MT7 was not visible post-transfer.

Rat OPC differentiation assays

All animal procedures were approved by the local Institutional Animal Care and Use Committee. OPC cultures were prepared as previously described [1,2]. Briefly, cortices from postnatal day 8 CD rat pups were collected (Charles River, Wilmington, MA). Tissue culture dishes were incubated overnight with goat IgG and IgM secondary antibodies to mouse (Jackson Immunoresearch, West Grove, PA) in 50 mM Tris-HCl

pH 9.5. Dishes were rinsed and incubated at room temperature with primary antibodies for Ran-2, Gal-C and O4. Tissue was diced and dissociated with papain (Worthington, Lakewood, NJ) at 37°C. Papain was inactivated with ovomucoid and cells filtered through a 40 µm nylon mesh (Fisher Scientific, Waltham, MA). Cells were pelleted at 300 x g for 15m and resuspended in a panning buffer (0.2% BSA in DPBS). Cells were incubated at room temperature sequentially on three immunopanning dishes: Ran-2 and GalC were used for negative selection before positive selection with O4. OPCs were released from the final panning dish using Accutase (Invitrogen, Carlsbad, CA). Accutase was inactivated with 30% FBS and OPCs centrifuged at 300g x 15m. Pellet was resuspended in complete media (DMEM, N2 and B27 supplements (Invitrogen, Carlsbad, CA), 5µg/mL N-acetylcysteine (Sigma, St Louis, MO), 5µM forskolin (MilliporeSigma, Burlington, MA), 50 ng/mL PDGF (Peprotech, Cranbury, NJ). OPCs were plated at 60,000 cells in 96-well Viewplates (Perkin Elmer, Waltham, MA) and maintained at 37°C, 5% CO_2.

OPC differentiation assay: The day after plating, complete media was replaced with complete media (without PDGF) and serially diluted compounds. Cells were incubated for 72h then fixed and immunostained. For assays using the MT7 probe, cells were incubated with 10nM probe for 1 hour, washed quickly in PBS, then fixed.

OPC differentiation assay (pulse dose): The day after plating, complete media was replaced with complete media (without PDGF) containing 300nM PIPE-307. PIPE-307 containing media was washed 2 times with complete media (minus PDGF) after 2, 8, 24, or 72h incubation. MT7 (300nM) was left on for 72h. At 72h the plate was fixed and processed for immunostaining.

For immunostaining, 4% paraformaldehyde fixative was washed 3x with DPBS. Cells were blocked with 10% goat or donkey serum in DPBS, incubated for 30-60 minutes at room temperature. After blocking, primary antibody was added, diluted in 10% goat or donkey serum, 0.2% Triton X-100 overnight at 4°C. Cells were washed 3x with DPBS and incubated with secondary antibody (all from Invitrogen either goat or donkey anti-rat Alexa 488, anti-rabbit Alexa 594, anti-goat Alexa 647) diluted in 10% goat or donkey serum, 0.2% Triton X-100 and 1:1000-2000 Hoechst 33342 (Invitrogen, Carlsbad, CA). Plates were imaged using a Nikon A1R confocal microscope at 20x and quantified. Primary antibodies used in these studies were rat anti-MBP (Millipore, Temecula, CA), goat anti-ChAT (R&D Systems, Minneapolis, MN), anti-rabbit PDGFRα (Invitrogen, Carlsbad, CA) with Hoechst 33342 counterstain (Invitrogen, Carlsbad, CA)

Rat cortical remyelination assay

The assay was performed as described in Lariosa-Willingham et al., 2022.[3] Briefly, forebrains were collected from embryonic day 18 rats, finely minced and digested in papain (Worthington, Lakewood, NJ) for 15 min, washed with 20% HBSS, then triturated with a P1000 pipet. Tissue was centrifuged (500 x *g*), the supernatant removed, the pellet resuspended in Neurobasal (Invitrogen) supplemented with N21 Max (R&D) and penicillin/streptomycin (Invitrogen), and plated at a density of 20,000 cells/well onto 96-well Viewplates (Perkin Elmer, Waltham, MA) coated with poly-D-lysine and laminin (Sigma, St Louis, MO). On day 4, media was replaced with myelination media [3] and different concentrations of PIPE-307 or 300nM MT7. Cells were fixed 9 days later with 4% paraformaldehyde (EMS, Hatfield, PA) for 15 minutes followed by three washes with DPBS. Cells were incubated in with rat MBP (Millipore, Temecula, CA), and mouse Tuj1 (Biolegend, San Diego, CA) antibodies diluted in 10% donkey serum containing 0.1% Triton X-100 (Fisher Scientific, Waltham, MA) overnight. Cells were then washed with 3 x DPBS followed by 1 hour incubation with secondary antibodies (anti-rat, Alexa488; anti-mouse, Alexa647) and Hoechst 33342 (1:2000) in blocking buffer. All secondary antibodies and Hoechst were produced in goat (Invitrogen, Carlsbad, CA) and used at 1:250. Images were acquired using a Nikon A1R confocal microscope and NIS-Elements software. Image analysis was performed using ImageJ. Only MBP segments that co-localized with Tuj1 axons were measured and the average myelin length per oligodendrocyte was calculated.

Mouse cortical brain slice culture

CD-1 mice at postnatal day 17 (Charles River, mixed gender) were used for the following experiments. All procedures were approved by the local Institutional Animal Care and Use Committee.

Evaluation of *Mbp* transcript in mouse brain slice culture: Brains were collected from P17 mice and placed into cold HBSS containing 20% FBS. Brains were bisected into left and right hemispheres and 250 µm McIlwain tissue chopper (Ted Pella, Redding CA). Only slices anterior to the hippocampus displaying a discrete corpus callosum were used. Approximately 12 slices are obtained per animal. Slices were laid on a 30 mm MilliCell organotypic culture insert in a 6-well culture dish containing 1.1 mL growth media (DMEM, 25% HBSS -Ca^{+2}/-Mg^{+2}, 25% heat inactivated horse serum, glucose (5 g/L), 25 mM ascorbic acid and penicillin/streptomycin). Slices were cultured *in vitro* for 72h with a 50% media change at 24h. After 72h, slices were demyelinated using media containing 0.5 mg/mL lysolecithin for 18h. Following demyelination, lysolecithin was replaced with media containing varying concentrations of PIPE-307 and treated for 72h. Slices were snap frozen and RNA extracted using the RNEasy Mini kit (Qiagen, Germantown, MD). Reverse transcription was performed using QScript (QuantaBio) followed by qPCR

with Perfecta PCR SuperMix (QuantaBio) on a StepOne Plus Thermocycler (ABI). Primers used were as follows: 18S rRNA F: GTCTGTGATGCCCTTAGATG, R: AGCTTATGACCCGCACTTAC; *Mbp*: F: CTATAAATCGGCTCACAAGG R: AGGCGGTTATATTAAGAAG. Cycling parameters: 95°C 30s, then repeat 45x: 95°C for 5s, 60°C for 15s, followed by melt curve analysis. Data was calculated using 2^{-ddCt}, normalizing to 18S rRNA and vehicle.

Immunohistological evaluation of MBP and Caspr in mouse brain slice culture: Brain slices were cultured as described above. After compound treatment, slices were fixed in 4% PFA for 30m, blocked in 10% donkey serum, 0.2% Triton X-100, followed by overnight incubation in primary antibodies in blocking solution. Antibodies used were MBP (rat, 1:500, Millipore, Temecula, CA), Caspr (rabbit 1:250 Abcam) Tuj1 (mouse, 1:500, Biolegend, San Diego, CA). Slices were washed with 3x15m PBS followed by incubation with secondary antibodies (anti-rat, Alexa488; anti-rabbit, Alexa568, anti-mouse, Alexa647, Hoechst counterstain) in blocking buffer. All secondary antibodies were produced in donkey (Invitrogen, Carlsbad, CA) and used at 1:250. Filters were excised from insert, mounted with Fluoromount (Sigma, St Louis, MO) on a microscope slide and coverslipped. Regions immediately superior to the corpus callosum were acquired using a Nikon A2 confocal microscope and NIS-Elements software. Image analysis was performed using ImageJ. MBP was expressed as MBP surface area normalized to Hoechst cell count; Caspr was expressed as the number of Caspr puncta normalized to Tuj1 intensity.

Human cortical slice culture

Human donor brain was received < 24 hours after death on wet ice (Tissue For Research Ltd, Biobankonline.com, Suffolk, UK). Upon receipt, the brain was immediately placed into-ice cold DPBS without calcium or magnesium. Cortical regions containing gray-white border were isolated then sliced on a McIlwain tissue chopper set at 400 µm thickness. Slices were laid on a 30 mm MilliCell organotypic culture insert in a 6-well culture dish containing plating media (DMEM (Invitrogen, Carlsbad, CA), B-27 (Invitrogen, Carlsbad, CA), 25 mM ascorbic acid (Millipore Sigma, St Louis, MO), sodium pyruvate (Millipore Sigma, St Louis, MO), Glutamax (Invitrogen, Carlsbad, CA), and penicillin/streptomycin (Invitrogen, Carlsbad, CA), 1 mM HEPES (Millipore Sigma, St Louis, MO). Slices were maintained in plating media for a minimum of 1 hour, then replaced with culture media. Half of the media was replaced every other day for 10 days. At day 10, culture media was replaced with culture media containing vehicle, PIPE-307 (300 nM) or MT7 (300 nM) and cultured an additional 9 days, replacing half of the media (containing compound) every other day. Slices were then processed for qPCR or immunohistochemistry.

Evaluation of *Mbp* transcript in human brain slice culture: After compound treatment, slices were immediately transferred to lysis buffer and RNA extracted using the RNEasy Mini kit (Qiagen). Reverse transcription was performed using QScript (QuantaBio) followed by qPCR with Perfecta PCR SuperMix (QuantaBio) on a StepOne Plus Thermocycler (ABI). Primers used were as follows: 18S rRNA F: GTCTGTGATGCCCTTAGATG R: AGCTTATGACCCGCACTTAC; *Mbp*: CTATAAATCGGCTCACAAGG R: AGGCGGTTATATTAAGAAG. Cycling parameters: 95 ^{o}C 30 s, then repeat 45x: 95 ^{o}C for 5s, 60 ^{o}C for 15 s, followed by melt curve analysis. Data was calculated using 2^{-ddCt} , normalizing to 18S RNA and vehicle.

Immunohistological evaluation of oligodendrocytes in human brain slice culture: After compound treatment, slices were fixed in 4% PFA overnight. Slices were gently lifted from the membrane insert and washed with at least five 15 minutes washes in DPBS containing 0.5% Triton-X 100 (0.5%-DPBS) followed by overnight incubation in primary antibodies in blocking solution. Antibodies used were CC-1 (mouse, 1:250, Millipore, Temecula, CA), Olig2 (rabbit 1:250 CellMarque, Rockland, CA). Slices were washed with at least five 15 minutes washes in 0.5%-DPBS followed by incubation with secondary antibodies (anti-mouse Alexa488; anti-rabbit, Alexa568, and Hoechst counterstain) in 0.5%-DPBS. All secondary antibodies were produced in goat (Invitrogen, Carlsbad, CA) and used at 1:250. Filters were excised from insert, mounted with Fluoromount (Sigma) on a microscope slide and coverslipped. 2 images per slice were acquired using a Nikon A1R confocal microscope and NIS-Elements software. Images were thresholded and counted using ImageJ.

In vivo *mouse receptor occupancy*

Female C57BL/6N mice were obtained from Envigo (Indianapolis, IN). All procedures were approved by the Pipeline Therapeutics Institutional Animal Care and Use Committee.

Mice were dosed with vehicle, PIPE-307, or clemastine. PIPE-307 was formulated in 0.5% methocel vehicle at 0.001, 0.003, 0.01, 0.03, 0.1, 0.3, 1.0, or 3.0 mg/mL for dose levels of 0.01, 0.03, 0.1, 0.3, 1, 3, 10, and 30 mg/kg, respectively. Clemastine was formulated at 1 mg/mL in 1% HPMC/0.1% Tween80 for a dose level of 10 mg/kg.

[^{3}H]-PIPE-307 was diluted to 9.8 µCi/mL in saline and administered via intravenous (IV) injection to the lateral tail vein at a dose volume of 5 mL/kg. Mice were euthanized by decapitation, blood collected, the brains rapidly dissected, forebrain and cerebellum isolated, and each region was weighed and placed into a 5 mL polypropylene tube. Tissues were diluted with a 10x volume of ice-cold binding buffer (50 mM

HEPES, 100 mM NaCl, 2 mM EDTA, pH 7.4) for a final dilution factor of 11-fold. Brains were then rapidly homogenized on wet ice (25,000 rpm, 7s). 350 µL of homogenate was filtered in duplicate of Whatman GF/B filters (GE Life Sciences, Marlborough, MA) which had been pre-wetted with 0.5% PEI prior to loading onto the Hoefer manifold. Filters were washed twice by applying 5 mL ice-cold wash buffer (50 mM Tris-HCl, 154 mM NaCl, 0.05% Tween 20, pH 7.4) to the manifold. Washed filters were removed from the manifold and placed into uncapped polyethylene scintillation vials to air dry. Following completion of the filtration of all samples, uncapped scintillation tubes were placed into an oven (~40-50°C) for ~ 30 minutes to ensure complete drying. Five (5) mL Ultima Gold F scintillation fluid (Perkin Elmer, Boston, MA) was added to each tube. Tubes were capped and allowed to equilibrate for approximately 30 minutes in the dark inside a Beckman LS6500 liquid scintillation counter. At the end of this equilibration period, samples were counted using a program specific for tritium detection. Total, non-specific (NSB) and specific binding were determined and M1 occupancy calculated.

Results are expressed as the percent occupancy of drug (i.e. percent inhibition of membrane-bound [^{3}H]-PIPE-307) and plotted as the mean ± SEM. The percent occupancy is derived by transforming the specific binding (total binding – non-specific binding) where non-specific binding is defined by the amount of membrane-bound counts remaining in the cerebellum, a region in which M1 expression is low. The resulting data are transformed to percent (%) occupancy by the formula (100-(drug group/vehicle group x 100) and plotted using GraphPad Prism (San Diego, CA).
Plasma and diluted brain homogenates were thawed and quenched with a 1:4 v/v ratio of acetonitrile with IS347 (Pipeline Therapeutics, San Diego, CA) as internal standard. Samples were centrifuged and supernatants injected for LC-MS/MS analysis of PIPE-307 or clemastine. Unbound plasma and brain concentrations of PIPE-307 were calculated using the mouse plasma protein binding and brain tissue binding free fractions $f_{u,plasma}$ = 0.09) and $f_{u,brain}$ = 0.068), respectively. Unbound concentrations of clemastine were not determined.

MOG-Induced EAE model

C57BL/6N female mice (10-13 weeks) were obtained from Envigo (Indianapolis, IN) and allowed to acclimate for at least 7 days. Mice were housed in an Innorack IVC mouse system (Innovive, San Diego, CA) with access to standard rodent chow and water *ad libitum*. Animals were subjected to a 12h light – 12h dark cycle. All procedures were approved by the Pipeline Therapeutics Institutional Animal Care and Use Committee.

On study day 0, EAE was induced using a MOG_{33-55}/CFA Emulsion PTX kit (Hooke Laboratories, part# EK-2110, Lawrence, MA). Briefly, per manufacturer's instructions, each mouse was administered a total of 0.2 mL emulsion delivered via dorsal subcutaneous injections of 0.1 mL each to the mid-scapula and the lower lumbar regions. Pertussis toxin (PTX) was prepared per lot-specific instructions and administered via intraperitoneal injection of 0.1 mL (200 ng) on day 0 (2h post-MOG administration) and again on day 1 (24h post MOG).

Mice were orally dosed (10 mL/kg) once daily for 22 days with 0.5% methocel vehicle, 3 mg/kg PIPE-307, 30 mg/kg PIPE-307 or 10 mg/kg clemastine. Each treatment group contained 12 mice, except for the age-matched non-MOG control group which contained 4 mice.

Body weights and clinical scores were recorded daily. The cumulative clinical score was calculated by summing the daily clinical scores for each animal over the duration of the experiment. Supportive care (subcutaneous fluids, Nutrical supplement) was provided to all animals with a clinical score greater than 3 and/or displaying a body weight loss greater than 10% from the previous day's weight.

Study design

Group	Dose level (mg/kg)	Endpoints
Control (non-MOG)	0	Body weight, clinical score, VEP, EM
Vehicle	0	Body weight, clinical score, VEP, EM
PIPE-307	3	Body weight, clinical score, VEP, EM
PIPE-307	30	Body weight, clinical score, VEP, EM
Clemastine	10	Body weight, clinical score, EM*

*VEP was not conducted on the clemastine group due to laboratory constraints

Clinical disability scoring system

Score	Observation
0	Normal
0.5	Limp distal tail
1	Limp/weak tail
1.5	Loss of righting reflex when placed in the prone position
2	Weak hind limbs, waddling gait, legs held close together when picked up, unilateral hind limb dragging
2.5	Bilateral hind limb paresis, dragging both hind limbs or tripping on hind feet
3	Unilateral hind limb or unilateral front limb paralysis
3.5	Bilateral hind limb paralysis, flat hindquarters
4	Mild fore limb paresis or partial front limb paralysis
4.5	Severe fore limb paresis, not alert
5	Moribund or complete bilateral fore and hind limb paralysis

VEP Recordings: VEP recordings were taken 21-24 days post MOG induction (for simplification, this will be referred to as a nominal day 21 VEP throughout this document). The duration of the procedure was performed under red light conditions to which the animals were habituated at least 1 hour prior to execution. Animals were anesthetized with an intraperitoneal injection of ketamine (75 mg/kg) and Xylazine (10 mg/kg). The pupils were dilated with 1.0% tropicamide, 1 drop per eye (Akorn Pharmaceuticals, Lake Forest, IL). One minute following application of the dilating agent, one drop of Genteal (Alcon Laboratories, Fort Worth, TX) was applied to each eye to maintain ocular moisture during anesthesia.

Binocular flash VEPs were conducted using a Celeris Model D430 system (Diagnosys , Lowell, MA). VEPs were recorded from each eye independently and simultaneously. Mice were placed onto the heated platform (37ºC) and instrumented with probes which were placed subcutaneously into the snout and dorsal occipital regions. The VEP execution started around 5-10 minutes after anesthetic injection. Each examination was comprised of at least 3 runs, with the following settings: pulse intensity 3 $cd.s/m^2$, frequency 1 Hz, on time 4 ms, pulse color: white-6500K, 100 sweeps per result. Average N1 latency was determined from best of 3 representative runs. Study termination was considered to be day 22. This was the last day that clinical scores were determined for all study animals. VEP recordings required several days and these occurred over days 21-24 (referred to as nominal day 21). Terminal tissue sampling occurred following the VEP recording. Oral dosing continued for any animals for which VEP recordings occurred on beyond day 22.

Electron Microscopy/*g*-ratio: On day 22-24, following VEP recordings, mice were deeply anesthetized with isoflurane anesthesia and whole body perfused with EM grade Karnovsky's fixative (3% glutaraldehyde, 2% paraformaldehyde in 0.1M phosphate buffer, pH 7.4). Tissues were stored in fixative until further processing for electron microscopy by the Charles River Laboratories (Durham, NC). Lumbar spinal cords were dissected and trimmed into 5 levels, rostral to caudal, and the most caudal segment was processed for EM analysis. Tissues (spinal cord or optic nerves) were post-fixed in osmium tetroxide, rinsed in distilled water (x2), dehydrated through an ethanol series (50%, 70%, 95%, 100% (2x)), transitioned through propylene oxide (2x), infiltrated in Epon-Araldite (1:1 EA:PO, 3:1 EA:PO, pure EA), and embedded in Epon-Araldite blocks. The blocks were polymerized overnight at ~85°C, and semi-thin sections (~ 1 µm, one per tissue) prepared and stained with toluidine blue using standard protocols. Semi-thin images were evaluated, and the most caudal region was identified as the region of focus for thin sections. Thin sections

of ~ 100 nm were prepared using standard protocols and the grids were imaged at 1500x on a JEOL JEM-1400+ transmission electron microscope fitted with an AMT 16 MP digital camera system. The direct magnification used was 1500x or 4000x for spinal cord and optic nerve, respectively. Digital images were analyzed using ImageJ (NIH, Bethesda, MD) to determine axon and myelin sheath circumference and g-ratios were calculated using the following equation: g-ratio=axon circumference/myelin sheath circumference.

Rat novel object recognition test

The rat novel object recognition test was conducted at Biotrial International Ltd, Rennes, France. Experiments were carried out using male Sprague Dawley rats (Charles River, Les Oncins-69592, France), weighing 200-280 g (~6-7 weeks) at the beginning of the experiments. Rats were housed in groups of 2-4 in polysulfone cages (floor area = 1500 cm^2) under standard conditions: room temperature (22±2°C), hygrometry (55±10%), light/dark cycle (12h/12h), air replacement (15-20 volumes/hour), water and food (SAFE, ref. A04) *ad libitum*. Rats were acclimated for > 5 days prior to experimentation. Vehicle and PIPE-307, at 1 mg/kg, 3 mg/kg and 30 mg/kg, were administered orally 2 h before the acquisition trial or 30 mg/kg 24 h prior. Saline or scopolamine were administered subcutaneously 30 min prior. Recognition index (RI) [RI=(TN-TF)/(TN+TF) x 100]. TN: Time spent in active exploration of the novel object; TF: Time spent in active exploration of the familiar object. RI represents the difference between the time spent exploring the novel object and the time spent exploring the familiar object during expressed as a percentage of the total time spent exploring both objects.

References

1. Mei F, Lehmann-Horn K, Shen YA, et al. Accelerated remyelination during inflammatory demyelination prevents axonal loss and improves functional recovery. *Elife*. Sep 27 2016;5doi:10.7554/eLife.18246
2. Lee S, Leach MK, Redmond SA, et al. A culture system to study oligodendrocyte myelination processes using engineered nanofibers. Nat Methods. Sep; 9(9):917-22.
3. Lariosa-Willingham K, Leonoudakis D, Bragge T, et al. An in vivo accelerated developmental myelination model for testing promyelinating therapeutics. *BMC Neurosci*. May 25 2022;23(1):30. doi:10.1186/s12868-022-00714-y
